# Exploring the Pharmacological Mechanisms of *Tripterygium wilfordii* Hook F against Cardiovascular Disease Using Network Pharmacology and Molecular Docking

**DOI:** 10.1155/2021/5575621

**Published:** 2021-08-14

**Authors:** Bingwu Huang, Chengbin Huang, Liuyan Zhu, Lina Xie, Yi Wang, Ning Zhu

**Affiliations:** ^1^Department of Anesthesiology and Perioperative Medicine, Wenzhou People's Hospital, The Second Affiliated Hospital and Yuying Children's Hospital of Wenzhou Medical University, Wenzhou 325000, Zhejiang Province, China; ^2^Department of Orthopedic Surgery, The Second Affiliated Hospital and Yuying Children's Hospital of Wenzhou Medical University, 109 Xueyuan West Road, Wenzhou 325000, Zhejiang Province, China; ^3^Department of General Practice, The Wenzhou Third Clinical Institute Affiliated to Wenzhou Medical University, The Third Affiliated Hospital of Shanghai University, Wenzhou People's Hospital, No. 299 Guan Road, Wenzhou 325000, Zhejiang Province, China; ^4^Department of Neurosurgery, The Wenzhou Third Clinical Institute Affiliated to Wenzhou Medical University, The Third Affiliated Hospital of Shanghai University, Wenzhou People's Hospital, No. 299 Guan Road, Wenzhou 325000, Zhejiang Province, China; ^5^Department of Cardiology, The Wenzhou Third Clinical Institute Affiliated to Wenzhou Medical University, The Third Affiliated Hospital of Shanghai University, Wenzhou People's Hospital, No. 299 Guan Road, Wenzhou 325000, Zhejiang Province, China

## Abstract

**Background:**

Tripterygium wilfordii Hook F (TwHF) has been used in traditional Chinese medicine (TCM) for treating cardiovascular disease (CVD). However, the underlying pharmacological mechanisms of the effects of TwHF on CVD remain elusive. This study revealed the pharmacological mechanisms of TwHF acting on CVD based on a pharmacology approach.

**Materials and Methods:**

The active compounds were selected from the Traditional Chinese Medicine Systems Pharmacology (TCMSP) database according to the absorption, distribution, metabolism, and excretion (ADME). The potential targets of TwHF were obtained from the SwissTargetPrediction database. The CVD-related therapeutic targets were collected from the DrugBank, the GeneCards database, and the OMIM database. Protein–protein interaction (PPI) network was generated by the STITCH database. Gene ontology (GO) and Kyoto Encyclopedia of Genes and Genomes (KEGG) pathway enrichment analyses were performed by R package. The network of drug-targets-diseases-pathways was constructed by the Cytoscape software.

**Results:**

The 41 effective ingredients of TwHF and the 178 common targets of TwHF and CVD-related were collected. Furthermore, AKT1, amyloid precursor protein (APP), mitogen-activated protein kinase 1 (MAPK), phosphatidylinositol 3-kinase catalytic subunit alpha (PIK3CA), and cellular tumor antigen p53 (TP53) were identified as the core targets involved in the mechanism of TwHF on CVD. Top ten GO (biological processes, cellular components, and molecular functions) and KEGG pathways were screened with a *P* value ≤0.01. Finally, we constructed the network of TwHF-targets-CVD-GO-KEGG.

**Conclusions:**

These findings demonstrate that the main active compound of TwHF, the core targets, and pathways maybe provide new insights into the development of a natural therapy for the prevention and treatment of CVD.

## 1. Introduction

Cardiovascular disease (CVD) is a collective term for cardiovascular and cerebrovascular diseases, which is the first cause of death in the world [1]. The burden of CVD is on the rise globally, especially in middle- and low-income countries (LMIC) [2, 3]. In 2013, the World Health Organization (WHO) proposed that countries should reduce premature mortality that related to noncommunicable diseases, including CVD, by 25% by 2025 [4]. Although Western medicines have made good progress in reducing the risk of cardiovascular events and total mortality, patients with long-term cardiovascular treatment still have difficult adherence that might lead to discontinuation of these drugs. This can be attributed to the adverse reactions caused by multiple pharmacologic agents and some drugs that beyond the affordability of LMIC [3, 5]. Traditional Chinese medicine (TCM), with thousands of years of history in China, has gained widespread clinical applications. In particular, TCM occupies a special position in their heart of the elderly. As a critical component of complementary and alternative medicine, TCM medications have been used for the prevention and treatment of CVD [6].

*Tripterygium wilfordii* Hook F (TwHF), also known as Leigongteng and Thunder God Vine, has possessed many pharmacological activities such as anticancer, anti-inflammation, antifibrosis, antiatherosclerosis, and antiautoimmune disorders [7–9]. Recently, several fundamental researches have indicated that low-dose TwHF can prevent cardiovascular diseases. Low-dose TwHF can improve the inflammatory reaction, reduce myocardial injury, and optimize acute coronary syndrome (ACS) rat's condition with inhibition of myocardial apoptosis [10]. TwHF extracts were shown to have cardioprotection effects by inducing the activation of Nrf2/HO-1 defense pathway, inhibiting the activation of NF-*κ*B pathway and reducing the expression of NLRP3 inflammasome [11–13]. In addition, the extracts can not only improve the vascular function in atherosclerosis, but also may help in the prevention of in-stent restenosis formation following endovascular treatment of lower-extremity artery disease [14, 15]. However, the underlying pharmacological mechanisms of the effects of TwHF on CVD remain elusive.

**Figure 1:**
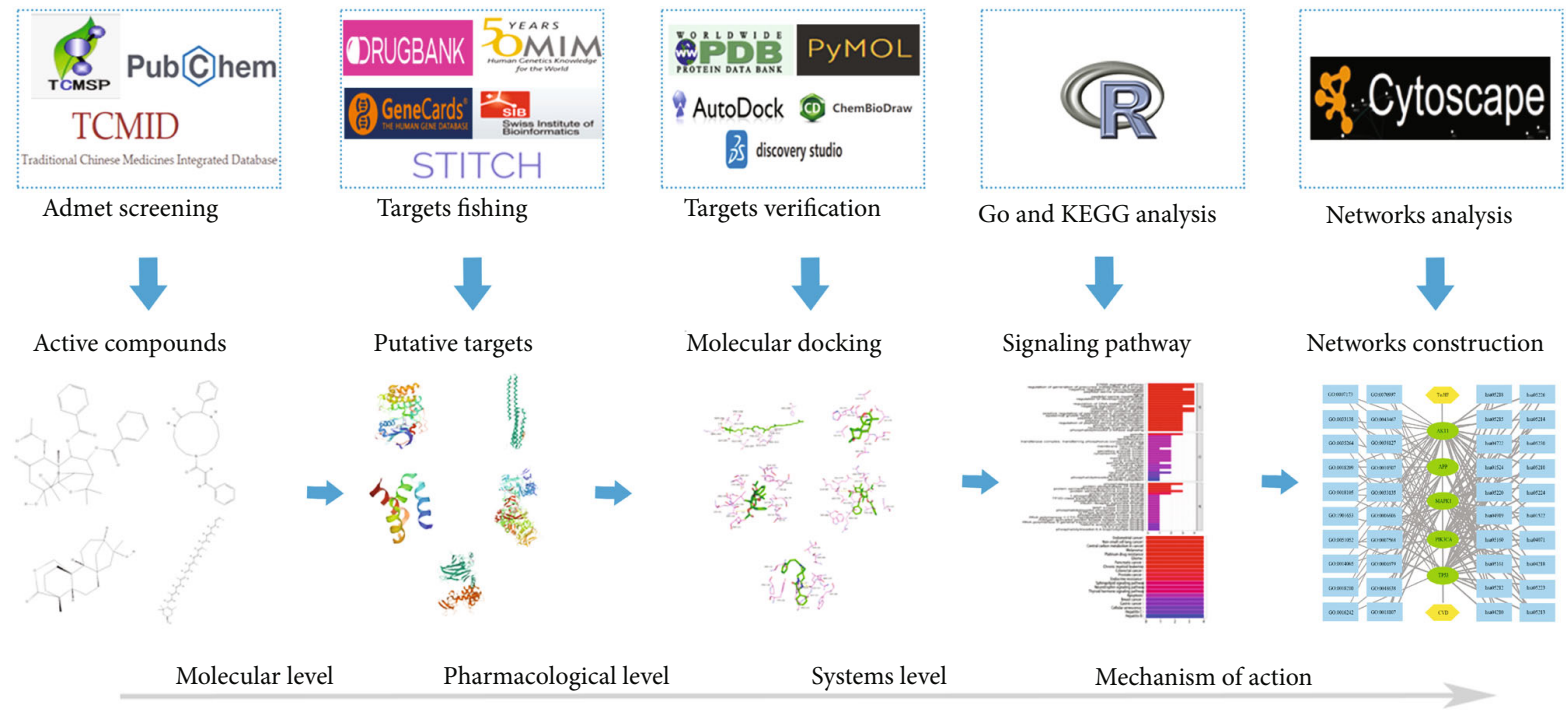
Network pharmacology for deciphering pharmacological mechanisms of *Tripterygium wilfordii Hook F* acting on cardiovascular disease.

Network pharmacology is an innovative way to analyze the complicated relationship between drugs and disease at the system level, which can provide clues for discovering new drugs [16]. This approach integrates and constructs the complicated networks among drug targets, disease targets, and biological processes [17]. It is possible to reveal potential drug-target-disease interactions and realize novel therapeutic application beyond the TCM application through network pharmacology [18]. In this study, target prediction, pharmacokinetic evaluation, molecular structure, biological function, and pathway analysis using many available public databases and bioinformatics tools have systematically elucidated the mechanisms of therapeutic effects of TwHF on CVD (Figure 1).

## 2. Materials and Methods

### 2.1. Active Component Screening

Traditional Chinese Medicine Systems Pharmacology database (TCMSP, https://tcmspw.com/tcmsp.php) is an efficient pharmacology resource, which can be used to assess the pharmacokinetics of TCMs or related compounds [19]. It can provide the absorption, distribution, metabolism, and excretion (ADME) properties of compounds, the main indicators of which are oral bioavailability (OB) and drug similarity (DL). OB is a reliable indicator to evaluate the intrinsic quality of drugs objectively, which represents to the speed and degree of absorbing drugs into the circulatory system. And DL represents the sum of the pharmacokinetic properties and safety of compounds, which is calculated by comparing the functional or physical properties of the compounds with those of the majority of known drugs [20]. In this paper, the compound name “Leigongteng” was inputted to the TCMSP database, and active ingredients with DL ≥ 0.18 and OB ≥ 30% were selected for subsequent analysis. Then, SMILES and PubChem ID of candidate components were collected by using the Traditional Chinese Medicines Integrated Database (TCMID, http://www.megabionet.org/tcmid/) [21] and the PubChem (https://pubchem.ncbi.nlm.nih.gov/) database [22].

### 2.2. Identified and Predicted Targets of TwHF

The targets of active components in TwHF were obtained from the SwissTargetPrediction (http://www.swisstargetprediction.ch), which is a free public resource used to accurately predict targets for bioactive molecules [23]. The therapeutic targets of active ingredients were predicted by inputting these components SMILES into SMILES string (s) and searching for their similar molecules. Within the range of “Homo sapiens,” high probability targets (probability *P* < 0.05) were collected after duplicate contents were removed.

### 2.3. Target Identification of Known Therapeutic Targets Acting on CVD

The CVD-related therapeutic targets were collected from the DrugBank (http://www.drugbank.ca) [24], the OMIM database (https://omim.org) [25], and the GeneCards database (https://www.genecards.org) [26]. DrugBank is a freely available network database, which provides molecular information about drugs, drug targets, drug effects, and drug interactions. OMIM database, a comprehensive web resource, is focusing on genes, genetic phenotypes, and their relationships. In addition, GeneCards is a public database that provides detailed information on annotated and predicted genes. With “cardiovascular disease” as the keyword, CVD-related targets were searched among the three databases.

### 2.4. Protein–Protein Interaction (PPI) Network Construction and Analysis

The identified targets were uploaded to the STITCH database v5.0 (http://stitch.embl.de/) [27] to build the protein–protein interaction network and clarify the functional and physical association between them. The protein interactions were limited to a combined score of 0.9 or higher. The core target genes were determined based on the criterion of combined score ≥ 0.9 and the number of interactions.

### 2.5. GO and KEGG Pathway Enrichment Analyses

GO analysis can supply gene product biological function information and divide candidate targets into various functional modules, including cellular components (CCs), biological pathways (BPs), and molecular functions (MFs) [28]. KEGG analysis can give functional meaning to genes at molecular or higher levels [29]. Enrichment analyses of GO of core target genes and KEGG were performed by using R (version 3.6.0 for Windows). By using a cut-off value adjusted to *P* < 0.05, the top ten GO enrichments and KEGG pathways were screened.

### 2.6. Construction of Network Relationships

Cytoscape is a free application software, which can transform biomolecular interaction networks into a versatile and interactive visualization framework [30]. The core targets of TwHF on CVD were constructed for KEGG-GO enrichment visualization by the Cytoscape (v3.7.1) software [31]. In the interactive network, the nodes include TwHF, CVD, and their core targets, GO and KEGG pathways. Then, the edges represent the interaction between them.

### 2.7. Molecular Docking

The crystal structures of target proteins were collected from the RCSB Protein Data Bank (http://www.pdb.org/) and decorated by removing the ligands and water motifs, adding hydrogen, and optimizing the mutation sites by the PyMOL (version 2.3). The 3D chemical structural formulas of key ingredients were collected from PubChem and energy minimized by using ChemBioDraw 3D (version 14). The sites, binding ability, and interactions between compounds and targets were analyzed by PyMOL, AutoDockTools (version 1.5.6), and Discovery Studio 2020 Clients [32, 33]. Autodock vina (1.1.2) was used to conduct docking between compounds and target proteins.

## 3. Results

### 3.1. Active Ingredient Screening

Total of 51 effective ingredients of TwHF that satisfied DL ≥ 0.18 and OB ≥ 30% were screened from TCMSP. But the structural formula of 41 effective ingredients of TwHF could be obtained from PubChem (https://www.ncbi.nlm.nih.gov/pccompound) for subsequent analysis (Table 1).

### 3.2. Target Identification of TwHF and CVD

Firstly, a total of 827 candidate targets of TwHF were downloaded from SwissTargetPrediction (Supplementary Table 1). Secondly, 76 known CVD-related targets were downloaded from the DrugBank database, 358 known CVD-related targets were downloaded from the GeneCards database, and 474 known CVD-related targets were downloaded from the OMIM database (Supplementary Table 1). Then, 802 CVD-related targets were identified by removing the repeated targets. Finally, the 178 common targets of the targets of CVD-related and TwHF were selected for subsequent analysis (Supplementary Table 1).

### 3.3. PPI Network Construction and Analysis

Firstly, the PPI network was generated by uploading these 178 identified targets to the STITCH database, and screening condition was limited to combined score ≥ 0.9. Then, AKT1, amyloid precursor protein (APP), mitogen-activated protein kinase 1 (MAPK), phosphatidylinositol 3-kinase catalytic subunit alpha (PIK3CA), and cellular tumor antigen p53 (TP53) were identified based on the number of interactions (Table 2). These five genes were considered the key putative targets involved in the effects of TwHF on CVD. The raw data (combined score ≥ 0.9) was shown in Supplementary Table 2.

### 3.4. GO and KEGG Pathway Enrichment Analyses

**Figure 2:**
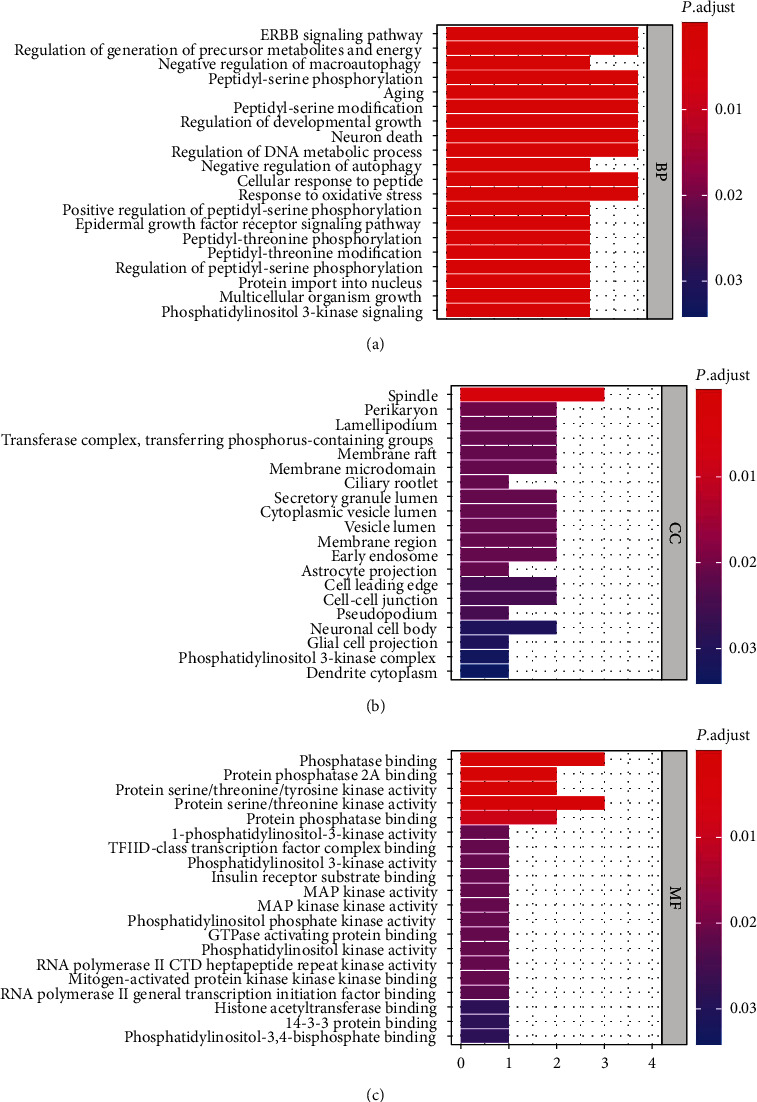
GO map of putative target genes. (a) Biological process categories. (b) Cellular component categories. (c) Molecular function categories.

**Figure 3:**
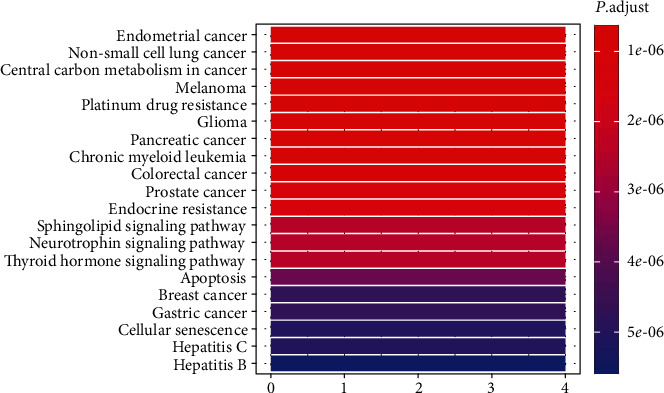
KEGG pathway analysis of putative target genes.

The 178 candidate targets were selected for GO and KEGG pathway enrichment analyses. The top ten GO analyses of biological process (BP), cellular component (CC), and molecular function (MF) categories were screened (Figure 2). As the results of GO enrichment, the enriched biological process categories were dominated by ERBB signaling pathway, regulation of generation of precursor metabolites and energy, peptidyl-serine phosphorylation, aging, peptidyl-serine modification, regulation of developmental growth, neuron death, regulation of DNA metabolic process, cellular response to peptide, and response to oxidative stress. CC analysis showed that the spindle was mainly accounted for the largest proportion. The enriched MF categories were dominated by phosphatase binding and protein serine/threonine kinase activity. The KEGG pathway analysis showed that these targets were mainly associated with cancer, melanoma, platinum drug resistance, glioma, chronic myeloid leukemia, endocrine resistance, sphingolipid signaling pathway, neurotrophin signaling pathway, thyroid hormone signaling pathway, apoptosis, cellular senescence, hepatitis C, and hepatitis B (Figure 3).

### 3.5. Construction of Network

**Figure 4:**
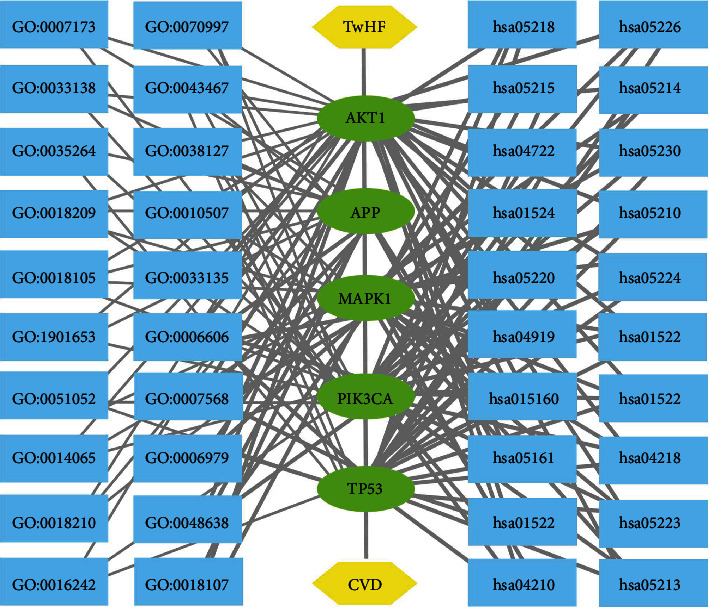
TwHF-targets-CVD-GO-KEGG network.

The network visualization of TwHF-targets-CVD-GO-KEGG was generated by using the Cytoscape software (Figure 4).

### 3.6. Molecular Docking

**Figure 5:**
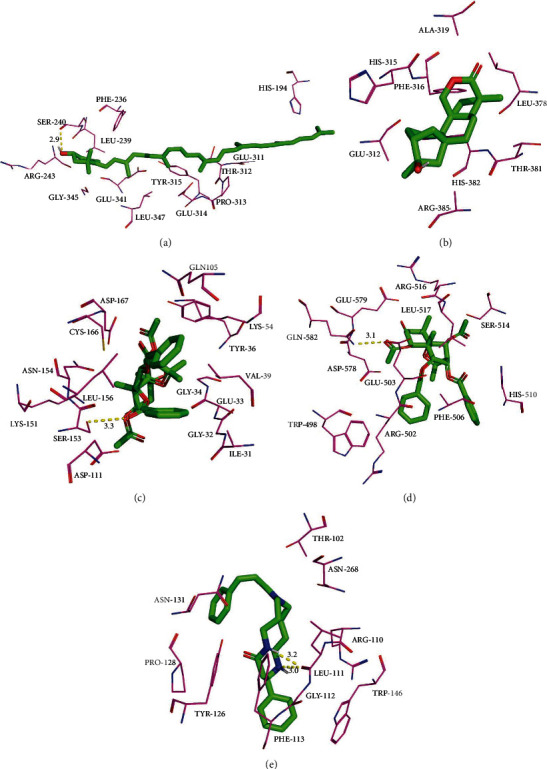
Molecular models of the binding of TwHF to the predicted targets (a) AKT1, (b) APP, (c) MAPK1, (d) PIK3CA, and (e) TP53 shown as 3D diagrams.

The crystal structures of potential targets, including AKT1 (PDB: 6CCY; 2.18 Å), APP (PDB: 5BUO; 2.31 Å), MAPK1 (PDB: 6SIG; 1.58 Å), PIK3CA (PDB: 4TTU; 2.18 Å), and TP53 (PDB: 6RZ3; 4.23 Å) were collected (Figure 5). Other detail of protein structure could be also found in RCSB Protein Data Bank. Figure 5 showed celaxanthin binds to AKT1 with a binding pocket consisting of SER-240 (2.9 Å); hypodiolide A fails to bind to APP without a binding pocket; triptofordin B2 binds to MAPK1 with a binding pocket consisting of SER-153 (3.3 Å) and ARG-155 (3.3 Å); triptofordin B2 binds to PIK3CA with a binding pocket consisting of GLN-582 (3.1 Å); celallocinnine binds to TP53 with a binding pocket consisting of LEU-111 (3.2 and 3.0 Å), ASN-131 (3.1 Å), and TYR-126 (2.9 Å).

## 4. Discussion

There is an urgent need to promote new drugs for CVD treatment because of the heavy burden of CVD and the poor efficacy and side effects of the currently used medicines. Network pharmacology was applied to reveal the interaction between medicines and targets of diseases, and it can comprehensively describe the complexity between drugs and diseases [33, 34]. Therefore, the use of network pharmacology uncovering multiple drug-target interactions may contribute to novel drug discovery in complex diseases such as CVD. TwHF exhibits therapeutic efficacy in preclinical models of CVD and has been identified in several studies [35–37]. In the present study, the underlying mechanism of the protective effects of TwHF on CVD was uncovered by a network pharmacology strategy. Therapeutic targets and signaling pathways were investigated by database screening, PPI network construction, and pathway enrichment analysis. Furthermore, to validate the specific interactions between core targets and CVD, molecular docking was conducted.

In this study, 41 active compounds of TwHF were determined based on ADME. Pharmacological analysis suggested that these active components may have protective effects against CVD. Nobiletin has been reported to attenuate hypoxia/reoxygenation-induced injury by the inhibition of oxidative stress and apoptosis in H9c2 cardiomyocytes, as well as myocardial ischemia and reperfusion injury in vivo [38, 39]. Triptonide ameliorates diabetic cardiomyopathy via mediating inflammation [40, 41]. Isoxanthohumol regulates vivo vascular proliferation and in vivo—the inflammatory crosstalk of vascular cells, contributing to the treatment of angiogenesis and inflammation-related diseases [42]. Stigmasterol blocked Ang II-induced aortic smooth muscle cell proliferation by the arrest of the cell-cycle and promoted apoptosis and ROS production [43]. Kaempferol attenuates cardiac hypertrophy and isoproterenol-induced heart failure in diabetic rats [44, 45].

Subsequently, the targets of TwHF and CVD were also identified. 178 common candidate targets between TwHF and CVD were selected. Finally, we screened 5 core candidate genes for further analysis. The interactive values and interaction indicate that these targets are closely contacted with other targets in “CVD-target PPI network” and are responsible for TwHF acting on CVD and the pathogenesis of CVD. As was well known, Akt signaling plays an important role in many processes of CVD pathology such as atherosclerosis, vascular remodeling, and cardiac hypertrophy. Several Akt inhibitors have been proven to be potential novel therapeutics for the CVD [46]. PIK3R1, MAPK1, and PIK3CA may modulate platelet activation and be involved in CVD [47]. Class I phosphatidylinositol 3-kinases (PI3Ks) are composed of a regulatory subunit (p85 regulatory subunit) and a catalytic subunit (p110 catalytic subunit) [48, 49]. The catalytic subunit p110*α* of PI3K is encoded by the gene PIK3CA, which regulates doxorubicin-induced cardiotoxicity [50]. Indeed, the compounded cardiovascular risk of PI3K*α* inhibitor use in breast cancer is particularly relevant given the prevalence of p110*α* gain-of-function mutations [51]. p38 mitogen-activated protein kinase (p38), extracellular signal-regulated kinase1/2 (ERK), and c-Jun NH2 terminal protein kinase (JNK) are major components of MAPK kinases, which control embryogenesis, differentiation, proliferation, and death [52]. The inactivation of JNK, p38MAPK, and ERK1/2 could block vascular smooth muscle cell proliferation and migration [53–55]. APP is associated with the adhesion of platelets to amyloid peptides and thrombus formation [56, 57]. It was reported TP53 can differentiate patients with left main coronary artery disease from healthy participants [58].

Top ten GO of each category (BP, MF, and CC) and KEGG pathways associated with TwHF acting on CVD were classified. These categories composed of the most key targets are considered specific and meaningful enrichment. The data showed that the major components were mainly related with multiple BPs, such as ERBB signaling pathway, regulation of generation of precursor metabolites and energy, peptidyl-serine phosphorylation, aging, peptidyl-serine modification, regulation of developmental growth, neuron death, regulation of DNA metabolic process, cellular response to peptide, and response to oxidative stress. It is reported that ERBB signaling can regulate cardiovascular development and multiple cardiac cell biology [59, 60]. Regulation of developmental growth and regulation of DNA metabolic process suggested TwHF acting on CVD through cardiovascular proliferation. However, BPs including precursor metabolites and energy, peptidyl-serine phosphorylation, neuron death, and cellular response to peptide have not been well investigated. Further studies should be conducted to determine the role of these BPs. The major components acting on CVD were also associated with spindle in terms of CC, which indicated TwHF acting on CVD was associated with regulation of the proliferation and differentiation of cardiovascular cells. The enriched MF categories were dominated by phosphatase binding and protein serine/threonine kinase activity. Various phosphatases promoted vascular remodeling and pulmonary arterial hypertension by modulating smooth muscle cell proliferation [61, 62]. Many serine-threonine kinases, such as P-21-activated kinases, PAKs, or RhoA/Rho-kinase, have been well demonstrated to promote the development of CVD [63, 64]. It was reported that there was a strong crosstalk between oxidative stress and various CVDs, including atherosclerosis, myocardial ischemia, ischemia reperfusion injury, and drug-induced cardiotoxicity [65, 66]. In addition, oxidative stress regulates multiple cardiovascular functions, such as cell proliferation and death [67]. KEGG pathway enrichment analysis showed TwHF may exert protective effects on CVD mainly by cancer pathways. Cancer and cardiovascular disease (CVD) share overlapping pathophysiology and risk factors as well as biological mechanisms [68]. Protein–protein interaction analysis has varied roles in driving and maintaining the growth of cancer and CVD [69, 70]. TwHF was also determined as an efficacy treatment of multiple cancers [71–73]. In summary, GO and KEGG pathway enrichments indicated that TwHF exerting protective effects on CVD probably through modulation of the proliferation of cardiovascular system cells.

According to the screening criteria of high OB, celaxanthin, hypodiolide A, triptofordin B2, and celallocinnine were chosen for the compound-ligand interaction analysis by molecular docking to validate the effects of TwHF acting on CVD. The results of molecular docking reflected that these active compounds possess suitable anti-CVD activity. However, to verify the active properties of TwHF and the molecular target genes of anti-CVD, further experimental studies need to be performed.

## 5. Conclusion

In summary, the network pharmacology method was performed to unveil the chemical basis and investigate the action mechanism of TwHF on CVD. Firstly, 41 active compounds of TwHF and 5 core target genes (AKT1, APP, MAPK, PIK3CA, and TP53) of TwHF against CVD were identified. Then, based on the analysis of GO and KEGG, the cancer pathway was found to be closely associated with the protective effect of TwHF on CVD. It provides a theoretical basis and a clue for the pharmacological mechanism study of TwHF on CVD in this study.

### 5.1. Limitation

There are some limitations to this study. Because of the collection of targets from databases, the predicted targets might be inaccurate and specific. Other values of compounds might be also inaccurate, such as the OB value of triptofordin B2, which is greater than 100%. These values also were predicted by chemometric method. Therefore, further experimental studies of these prediction results are needed to validate the potential applications.

## Figures and Tables

**Table 1:** A list of the final selected compounds from TwHF for network analysis.

Molecule ID	Molecule name	OB (%)	DL
MOL003233	Triptofordin B2	107.71	0.76
MOL003209	Celallocinnine	83.47	0.59
MOL003188	Tripchlorolide	78.72	0.72
MOL003206	Canin	77.41	0.33
MOL003225	Hypodiolide A	76.13	0.49
MOL003279	99694-86-7	75.23	0.66
MOL003208	Celafurine	72.94	0.44
MOL003244	Triptonide	68.45	0.68
MOL005828	Nobiletin	61.67	0.52
MOL002058	40957-99-1	57.2	0.62
MOL003217	Isoxanthohumol	56.81	0.39
MOL003224	Tripdiotolnide	56.4	0.67
MOL000211	Mairin	55.38	0.78
MOL003187	Triptolide	51.29	0.68
MOL003280	Triptonolide	49.51	0.49
MOL003185	(1R,4aR,10aS)-5-hydroxy-1-(hydroxymethyl)-7-isopropyl-8-methoxy-1,4a-dimethyl-4,9,10,10a-tetrahydro-3H-phenanthren-2-one	48.84	0.38
MOL003248	Triptonoterpene	48.57	0.28
MOL003196	Tryptophenolide	48.5	0.44
MOL003211	Celaxanthin	47.37	0.58
MOL003267	Wilformine	46.32	0.2
MOL003184	81827-74-9	45.42	0.53
MOL011169	Peroxyergosterol	44.39	0.82
MOL000449	Stigmasterol	43.83	0.76
MOL003245	Triptonoditerpenic acid	42.56	0.39
MOL000422	Kaempferol	41.88	0.24
MOL003231	Triptoditerpenic acid B	40.02	0.36
MOL003232	Triptofordin B1	39.55	0.84
MOL000296	Hederagenin	36.91	0.75
MOL000358	Beta-sitosterol	36.91	0.75
MOL003222	Salazinic acid	36.34	0.76
MOL003189	Wilforlide A	35.66	0.72
MOL003229	Triptinin B	34.73	0.32
MOL003266	21-Hydroxy-30-norhopan-22-one	34.11	0.77
MOL003238	Triptofordin F1	33.91	0.6
MOL003239	Triptofordin F2	33.62	0.67
MOL003278	Salaspermic acid	32.19	0.63
MOL003235	Triptofordin D1	32	0.75
MOL003241	Triptofordin F4	31.37	0.67
MOL003242	Triptofordinine A2	30.78	0.47
MOL003236	Triptofordin D2	30.38	0.69
MOL003210	Celapanine	30.18	0.82

**Table 2:** A list of the key putative targets involved in the effects of TwHF on CVD.

Name	The number of interactions
PIK3CA	50
AKT1	36
APP	34
TP53	34
MAPK1	31

## Data Availability

The data used to support the findings of this study are included within the article and the supplementary files.

## Supplementary Materials

Supplementary Materials Known targets of TwHF and known therapeutic targets acting on CVD were shown in Supplementary Table 1. Known protein interactions of TwHF against CVD (combined score ≥ 0.9) were shown in Supplementary Table 2.
