# Supplementary material for: Exploring the Pharmacological Mechanisms of Tripterygium wilfordii Hook F against Cardiovascular Disease Using Network Pharmacology and Molecular Docking
Source: Biomed Res Int. 2021 Aug 14;2021:5575621. doi: 10.1155/2021/5575621 (PMC8382521; doi:10.1155/2021/5575621)
Supplement: Supplementary Materials — Known targets of TwHF and known therapeutic targets acting on CVD were shown in Supplementary Table 1. Known protein interactions of TwHF against CVD (combined score ≥ 0.9) were shown in Supplementary Table 2. [file 5575621.f1.zip › Supplementary Table 1.pdf]

Known targets of TwHF
Swiss Target Prediction

CYP19A1
G6PD
HSD17B3
CDC25A
SERPINA6
SHBG
AR
GABBR1
HSD11B1
NPC1L1
POLB
GPBAR1
AKR1B10
VDR
HSD11B2
NR1H4
GABRA2
GABRB2
GABRG2
FABP4
PPARA
FABP3
FABP5
PPARD
FABP2
SLC22A6
UGT2B7
PTGFR
FNTA
FNTB
HAO1
FFAR1
KDM2A
PHF8
KDM5C
CDC45
PTPRC
CNR2
CDC25B
PLG
CCR1
PTGER2
PRKCA
ALOX5AP
SHH
CACNA2D1
STS
NR1H2
PTPN1
ESR1

ESR2
KCNA5
KCNA3
SLC6A12
GSTK1
LTA4H
CHRNA7
TRPM8
CA4
MDM2
GNRHR
P2RX3
NPY5R
TRPV1
JAK2
AGTR1
PRKCD
SRD5A1
HSD3B1
TACR1
HMGCR
EPHX2
PIK3CB
ACACB
SLC5A1
PRKCG
RPS6KA5
GLRA1
KCNH2
KDR
HTR2B
PTAFR
MAPK14
F2R
AKR1C3
TBXAS1
RXRA
DUT
PYGL
PLA2G7
PIM1
SLC6A2
TACR2
PIM3
JAK3
C5AR1
APP
PDE2A
MAPK13
PDE10A
MGLL
FASN

SGK1
CYP51A1
NR1H3
RORC
CYP17A1
SREBF2
CYP2C19
RORA
SLC6A4
BCHE
CHRM2
NR1I3
ACHE
PTGER1
PTGES
CES2
DHCR7
SQLE
PTPN6
NOS2
NR3C1
PPARG
CHRM4
DRD2
PREP
PTGER4
IDO1
ATP12A
PTGIR
DHCR7
EBP
TERT
FABP1
SIGMAR1
ADORA3
MAPK3
PTPN11
MDM4
TTK
PTPRF
PLA2G1B
ACP1
AVPR2
AVPR1A
SCD
IGF1R
SRC
ALK
BACE2
CCR5
ADRA2C
PRKCB

INCENP
AURKB
PRKCE
PRKCQ
PTGER3
PTGDR
GRM2
TOP1
HIF1A
CNR1
OPRM1
CD81
PTPN2
PDE4D
LTB4R
ALOX5
PTGS2
NR3C2
FAAH
PTGS1
SLC6A3
SRD5A2
PRKCH
PGR
PTGDR2
AMPD2
GRIK1
GRIK2
IL6
GLUL
MMP3
MMP1
MMP2
BACE1
EDNRB
EDNRA
SAE1
UBA2
CES1
POLA1
HSF1
MMP13
ITGB1
ITGA4
UBLCP1
SLC10A2
SLC10A1
MME
S1PR2
PLCG1
IKBKB
EPHA2

ACE
ST3GAL1
PPM1B
PPP1CC
TNF
HDAC6
HDAC1
PLA2G4A
CCKBR
ITGA2B
ITGB3
OPRD1
RARG
RXRG
BRS3
TYMS
RXRB
XIAP
HCAR2
EPHB2
EPHA5
EPHA4
EPHA8
EPHA6
EPHA7
EPHB3
EPHA3
EPHB1
EPHB4
EPHA1
EPHB6
THRA
THRB
PGGT1B
FNTA
ITGAV
MCL1
PPP2CA
DGAT1
FDFT1
CMA1
CYSLTR1
MAPK10
CPB2
ALOX15
ALOX12
CALCRL
SLC29A1
OXTR
MMP9
CYP1B1
IGFBP6

IGFBP4
IGFBP5
IGFBP2
IGFBP3
IGFBP1
NOX4
XDH
CA2
CA7
CA12
SLC37A4
HSP90AB1
CETP
MAPT
KDM4E
TOP2A
INSR
PTPRS
GLO1
MYLK
DAPK1
SYK
ABCC1
PIK3CG
MPG
APEX1
EGLN1
SLC22A12
HSP90B1
DUSP3
NQO2
PIK3CA
CDC7
EGFR
F2
DRD4
MPO
PIK3R1
ADORA2A
CA1
GSK3B
HSD17B2
CA3
CA6
CDK1
CA14
CA9
CSNK2A1
CXCR1
CAMK2B
CA5A
ABCG2

AKR1C2
AKR1C1
AKR1C4
CA13
ADORA1
AKR1A1
KDM4C
KIT
GPR35
TNNC1
TNNT2
TNNI3
CDK5R1
CDK5
CCNB3
CDK1
CCNB1
CCNB2
CDK6
CDK2
ARG1
TYR
HSD17B1
AHR
ESRRA
MKNK2
MKNK1
THRB
RCE1
RARB
RARA
ECE1
AMPD1
AMPD3
GSK3A
BCL2L2
TP53
MMP8
CCR2
TBXA2R
BRD4
TKT
CSF1R
FLT1
OPRL1
SLC6A9
PDE7A
AKR1B1
CSNK2A2
AURKA
TTL
DRD5

CDC25C
PTK2
HAO2
IMPDH1
MTOR
CTSA
CPA1
PDE5A
DPP4
HTR2A
SLC6A1
RPA1
ADAMTS5
MMP14
ITGB7
GCK
RBP4
CDK2
CCNA1
CCNA2
GRIA2
TGFBR1
KDM4A
ABCB1
MMP12
CTSD
REN
AVPR1B
NCOR2
HDAC3
HDAC8
CASP8
CASP1
CFD
F10
CAPN1
CDK9
ADAM17
CCNE1
CDK9
CCNT1
CDK7
CDK4
CTSK
CTSS
CTSL
CTSG
ADAM10
CTSV
CTSB
CCNB1
CDK7

CCNH
IMPDH2
CAPN2
PSMB9
HTR1A
CASP3
HTR7
MAP2K1
PAK4
PAK1
CCND1
CHRM3
DRD3
IRAK4
STAT3
PSMB5
JAK1
TYK2
SCN9A
SEM1
PSMD8
PSMD4
PSMD13
PSMD7
PSMD6
PSMD11
PSMD12
PSMD3
PSMD2
PSMC5
PSMC3
PSMC6
PSMC4
PSMC1
PSMC2
ADRM1
PSMD14
PSMD1
PSMB7
PSMB4
PSMB3
PSMB11
PSMB10
PSMA8
PSMA7
PSMA6
PSMA5
PSMA4
PSMA3
PSMA2
PSMA1
PSMB6

PSMB9
PSMB8
PSMB5
PSMB1
PSMB2
ADRA1A
TAB1
MAP3K7
TNK2
HTR2C
RASGRP3
GBA
FKBP1A
ADK
BDKRB1
PSMB8
NTRK1
PDE6A
BRAF
PAK3
PAK2
ADRB1
VHL
ELOC
ELOB
TK2
LIPG
PDE4A
SLC5A2
KARS
CCNE2
CCNE1
CXCR2
PIM2
GABRA5
GALR1
GALR2
MCHR1
PRKDC
ATP6AP1
TMIGD3
HK2
HK1
CSNK1A1
PSMB1
PIK3CD
P2RX7
PLEC
CASP7
SIRT2
MAP3K9
TRPA1

F11
PIK3CA
FLT3
PFKFB3
MAP3K1
ADCY1
CASR
DYRK1A
MAPK1
PSEN2
PSENEN
NCSTN
APH1A
PSEN1
APH1B
SCARB1
BRPF1
RPS6KB1
SLC16A1
SLC2A1
CTSE
AURKC
BCL2
PDE4B
CYP26A1
PIN1
CPT1A
CPT1B
AGTR2
FGFR1
CPT2
CACNA1G
BMP1
CPB1
PDGFRB
ITGB6
PTPN22
OGFRL1
PRSS1
CTRC
EPAS1
PLA2G2A
NLRP3
JUN
CYP24A1
MTNR1A
MTNR1B
TLR9
MC5R
F2RL1
ABL1
BCL2L1

LGALS3
LGALS9
ERBB2
HCRTR2
HCRTR1
ABCC9
NFKB1
ATR
RELA
ROS1
F9
BDKRB2
BRD2
BRD3
LGMN
SMO
PPID
PPIB
PPIG
HSP90AA1
TRHR
PLAT
TSPO
UGCG
ROCK2
TSHR
MAPK8
GRM5
NTRK2
LDLR
CRHR1
MET
PANK3
RPS6KA3
NPBWR1
GABRB3
GABRA3
GABRG2
GABRA1
TK1
CCNC
CDK8
PDE9A
GPR139
GRM1
PDE1C
EPHX1
S1PR3
CYP11B1
HLA-DRB1
KCND3
OPRK1

LIPA
SLC18A3
RHOA
TRPV4
PABPC1
PRCP
GABBR2
RASGRP1
PPP5C
PPP1CA
RAPGEF4
GCGR
KCNQ2
PAM
KCNQ1
PKM
PRMT3
HSD17B7
CHRNA4
CLK4
LCK
HDAC2
MAPKAPK2
CSNK1G1
CYP11B2
HRH3
CSNK1D
HRH4
F2RL3
FLT4
PIP4K2C
SLC27A1
HTR6
YES1
GSTO1
HIPK1
DBF4
HNF4A
TRAP1
NR4A1
PGGT1B
OGT
DHFR
LRRK2
PDE3A
GSTM1
ATP2A1
MAOB
PCSK7
VAV1
IARS
NFKBIA

ADRA1D
MGMT
ADORA2B
GLI1
IL1B
LPL
MMP7
KDM4B
KDM5B
MAPK9
EWS-Fli1
EIF2AK3
ALPL
ACPP
ATP1A1
PARP1
PTPA
ELANE
SLC5A4
SLC28A3
ALDH2
TAS2R31
EIF4A1
CBR1
LGALS1
CA5B
LGALS7
TDP1
CHIA
FOLH1
HRAS
AMY2A
ALB
TARS
SLC28A2
FHIT
BTK
TRPM2
SERPINE1
FUT6
NRP1
DOT1L
CASP6
METAP2
CHEK1
ODC1
PLA2G5
PLA2G10
KLK1
KLK2
ESRRB
SPHK2

SPHK1
MMP16
MMP15
MMP26
NAAA
PDK1
CDK3
WEE1
RET
DRD1
CLK1
DYRK1B
DNM1
F3
TNKS2
TNKS
HDAC11
PDF
HDAC10
HDAC5
NEK1
EEF2K
ADRA2A
ADRA2B
HDAC7
CAPN1
CAPNS1
FCER2
ADRA1B
PITRM1
CACNA1H
PRKCZ
RAC1
PTK2B
MIF
PASK
HPSE
KIF11
ITK
ADCY10
ELAVL1
GABRA1
GABRB2
HTR1B
GPR55
CXCR3
CREBBP
LIMK2
SAE1
EP300
KAT2B
PTK6

TRPC6
TRPC3
PDE3B
GC
PLA2G2C
MAP3K20
AKT1
ADRB2
MOGAT2
EZH2
ENPP2
CCND3
CDK4
CCND2
IRAK1
AXL
MAP4K2
MERTK
LTK
TACR3
AKT2
RORB
HTR1D
CCKAR
FAP
CX3CR1
PGA5
MAP3K11
MAP3K10
LIPC
PRSS3
SLC18A2
NR1I2
NR5A1
CHRNA3
CHRNB4
NR5A2
CHRNA4
CHRNB2
CHRM1
HPGD
HRH1
ALDH1A1
CHRM5
NPSR1
LSS
MGAT2
S1PR1
SOAT1

Known therapeutic targets acting on CVD

OMIM	Drugbank	GeneCards
RAI	PTGS1	TTN
HEY2	PPARG	IL6
HSPB7	PTGS2	APOE
MFS	FFAR1	TNF
DGS	FADS1	FBN1
WBS	SLC8A1	ACE
FBN1	ACSL4	LMNA
NF1	PPARD	INS
CRP	TRPV1	IL10
PXE	ACSL3	NOTCH1
ACE	ACE	GLA
CYP2C19	GABRA1	MTHFR
APOE	GABRA2	VWF
HOS	GABRA3	APP
HALP1	GABRA4	MYH7
IL18	GABRA5	CRP
HP	GABRA6	TGFB1
GATA6	GABRB1	SCN5A
CMH1	GABRB2	SNCA
PON1	GABRB3	TP53
ESR1	GABRD	VEGFA
TBX1	GABRE	APOB
IL6	GABRG1	NOS3
ABCA1	GABRG2	GBA
CSTLO	GABRG3	TLR4
KDR	GABRP	F2
PPARG	GABRQ	CD40LG
HMGCR	ADRA2B	IFNG
CITED2	CHRM1	IL1B
UCP2	CHRNA2	APOA1
ECTOL1	P2RY12	FLNA
IGHD1A	NR3C1	PPARG
SGS	ESR1	SERPINE1
NOTCH1	CHRM2	MAPT
BCAR1	NR1I2	PSEN1
GACI1	HMGCR	CCL2
FABP2	ADRB1	NOD2
PTEN	ADRB2	LEP
APOA1	ADRB3	RYR2
AAAS	HTR2C	MYH6
ADM	SLC6A4	HLA-DRB1
HABP2	GLRA1	CTLA4
NIDDM	SLC6A2	FBN2
WFS1	PLA2G2A	TTN-AS1
MELAS	NOS2	DMD
FTHS	NOS3	IGF1
CMD1DD	MAPK14	ABCA1
GACI2	MAPK12	PKD1
ATFB14	MAPK11	MYBPC3
TNCY	MAPK13	RET

ARCL2D	ALOX5AP	ALB
PUFAQTL1	ADRA1A	MYH11
NKX2-5	ADRA1B	KCNQ1
SCN2B	ADRA1D	ELN
ADK	CA1	CACNA1C
ALOX5	CA2	MPO
NCOA3	CA4	EDN1
NSD1	SQLE	TNFRSF11B
APOBR	PRKCB	AGTR1
MIR33A	PRKCA	SOD1
VPS54	CSNK2A1	ICAM1
MYH11	SYK	AKT1
PTPRB	CA12	REN
TBX20	CA9	PON1
ORTHYP1	CA3	KCNH2
MMP9	CA5A	LPL
GOT1	CA5B	CCR6
FOXC1	CA6	PRKAG2
MTTL1	CA7	LAMP2
GUSB	CA14	STAT3
CTHM	PRKACA	GATA4
CX3CR1	PPARA	JAG1
GBA	F2R	NPC1
ACDMPV	CACNA1C	PRNP
ITGB1	MME	BDNF
HDLCQ12	NCOA1	NKX2-5
TH		MMP2
ABCC6		TGFBR2
PVNH1		GAA
NR1H2		PRKN
FADD		COL3A1
ARCL3A		CST3
HFM		TTR
BDKRB2		LDLR
PLXND1		PKHD1
EDSSPD1		IL1RN
HSAN3		SMAD4
ACH		NPPA
MSPKA		ESR1
ALGS2		RYR1
VSD1		ABCA4
NPHP16		F8
MCAHS1		VDR
OI18		FAS
ARHI2		ANK2
MEOX2		NOS2
LIMK1		SERPINC1
SAA1		CAV3
MBL2		AGT
CRKL		ACTA2
ACADS		THBD
RERE		CETP

FBN2	HLA-B
ARMC4	TLR2
NPY1R	SMAD3
PRKG1	DES
FADS1	TGFB2
EDSCV	MMP1
COL15A1	CAT
ALDH18A1	ADIPOQ
THOP1	EGFR
EPHB4	COMT
NLN	TNNT2
ARMD1	TBX5
LPL	JUP
PPARGC1A	F5
AOVD1	GDNF
VEGFA	PKD2
CHDS7	PSAP
AGTR1	GFAP
EFEMP2	TGFBR1
SSKS	KIT
HTR2B	GJB1
NR1H3	TNFRSF1A
RTSC1	LRRK2
AOMS1	PTPN11
NR3C1	SMPD1
ANGPTL3	CDKN2A
ECYT1	MPZ
FXS	FN1
HJCYS	DSP
LS	LCAT
DFNB68	PTPN22
HTGTI	KRAS
CPPB2	POMC
GPHYSD1	HFE
LDLCQ8	PTEN
ATFB5	SPP1
PRDX2	CFTR
THRA	COL5A1
ARRB1	EDNRB
INVS	TNNI3
LTBP3	IL2RA
ACADM	MTOR
CYBA	HMOX1
TET2	MME
TRIM72	SLC17A5
NTF3	MMP3
TSTD1	TNFRSF1B
IFNA1	CXCL8
SVAS	RETN
COL18A1	MMP9
GPX1	HRAS
PIK3CG	ENG

CYP19A1	COL2A1
KIDINS220	EPO
DTGA1	GGT1
LDS1	NPPB
GDF15	MUC1
NIHF	JAK2
SMAD6	B2M
ARCL1B	LRP5
KLK1	CYBB
ADAP2	CTNNB1
ARCL1C	NLRP3
TGFBR2	PRTN3
MTHFR	CP
HTX5	TBX1
COL3A1	STAT1
CRHR2	TH
EVC	GJA1
NXD	VCP
MCLDS	ELANE
ANHD	FGF23
CMD1D	FOXP3
ARMD4	MIR21
VSD2	NF1
CILD23	HTT
SSS2	PLG
MC1DN13	TRPV4
CMH21	GNAS
HSD11B1	COG2
UGT1A1	CBS
NPPA	POLG
MMP3	APOC3
ADAM10	NGF
EPHB6	AGL
CHD7	SOD2
NF1	TNFSF11
MIR33B	CFH
SLC6A2	CCR5
IQCE	CD40
INSR	HLA-A
STWS	MAPK1
DES	WT1
HBG2	IL13
TFAP2B	CYBA
PRKCH	LIPC
CELF2	MGP
CMD1V	FGFR1
FRNS	COL1A1
MESP1	ABCB1
FHBL2	ATP7B
MFN2	EGF
AOVD2	PLOD1
TBXA2R	F7

TUBB1	ADRB2
NS8	HBB
ACE2	BRCA2
GDF1	MEFV
BTHS	LEPR
ELN	DSC2
FOXC2	MED12
ADM2	VHL
NIDDM1	IL18
MEND	BRAF
STL1	GHRL
HHF2	PMP22
XPB	NAGLU
VSD3	SKI
LDS5	IL23R
PKD1	HCN4
GRD1	FGFR3
FGQTL2	FLT1
GPR14	ECE1
VLDLR	VCL
NPR1	BRCA1
THBD	PCSK9
CMKLR1	PDGFRB
NSD2	PIK3CA
DAND5	NR3C2
MIR21	ERCC6
FGD6	MYLK
HNRNPA1	TSC2
PLEKHA1	MIR146A
IL4	SLC6A3
ABDS	C3
EPOR	LDB3
IGF2	MECP2
NRP1	SLC6A4
WASF2	PTGS2
SEMA3C	IGF2
KLF2	BAG3
GATA4	ENPP1
SELP	CD36
LDS2	MYC
EGFL7	TF
GSD2	NR3C1
SOX18	FGFR2
CBS	MTR
LDS3	TERT
ASIP	HSPD1
TRNT1	SOX10
XLHR	FGA
ISL1	ACTC1
MAPK7	HEXA
MKL2	CAV1
LQT1	G6PD

AEACEI	IL4
CFC1	EDNRA
TMAU	ADA
NLSDM	IL2
CORD16	PLA2G7
WITKOS	SYP
CPPB1	DRD2
RLS1	PTH
FFS	PRODH
GCKR	BSCL2
HCCS	ACP5
CD40	COL5A2
TYMS	F9
SPRY1	UMOD
HEY1	PSEN2
CYP17A1	TGFB3
ARMS2	APOA5
CYP21A2	SHH
LEP	AR
SELENOS	CCND1
ICAM1	MIR155
SINO	LOX
CFH	CDH1
IL11	IL12B
NDUFA2	ATM
BRIP1	SELE
LDLR	HSPG2
UQCRC2	SERPINA3
CCM2	EDN3
CHTD6	ARSA
ADRB1	RBP4
OI1	HBA1
PLA2G7	CTSD
PCSK9	NTRK1
IGHD4	LPA
PTGS2	ITGB3
ANKS6	HTR2A
AOS1	COL4A1
TBX2	XDH
MAP3K3	HLA-DQB1
APELA	CYP11B2
WHS	LRP1
MRLS	CASP8
HSCR1	DYNC1H1
CMD1M	GATA6
HDLCQ6	CCN2
THCYT3	SELP
CMD1R	TSHR
LIDLS1	CASR
MVP1	CRYAB
CRH	ADAM17
FOLH1	NDP

CD40LG	MBL2
DST	TNFRSF11A
PDGFB	SOX2
NEDD4L	BCHE
CHRD	ABCC8
DBH	TBX20
ARID5A	CEP290
FOXO3A	INSR
MAS1	PLAU
NPHP3	PROM1
SCD	IL17A
VETD	GH1
FGA	HMGCR
NOTCH2	ABCC9
TGFBR1	RUNX2
SLC2A9	HLA-DQA1
POU1F1	BMPR2
MTCO1	PON2
EDN1	HGF
NGPS	LTA
DA9	TLR3
PWS	PRKG1
JAG1	COL1A2
VAV3	BCL2
THAS	CYP17A1
IGF1	TG
OLR1	ESR2
PITA1	MEN1
NODAL	CD28
B3GAT3	LIPA
HEG1	BMP2
AGOTC	GLI3
CLS	FMR1
LPHDST	NOTCH3
ARH	TEK
AOS2	AIRE
HSAN6	PRKCSH
CTRCT16	MIR126
NEDBEH	KCNJ5
VVS	CRYAA
FGF1	MT-CO1
NR3C2	TRPM4
AR	PYGM
CAPNS1	CXCL12
DYNLL1	EMD
GP1BA	FLT4
LIPG	GP1BA
NEK8	SLC2A10
CHTOP	
SCN1B	
SERPINE1	
ELOVL4	

COL21A1
MDDGB2
FGB
MAPK14
ESX1L
FBXW7
ENPP1
ATP5F1C
APOB
HGPS
MYHRS
IL10
TNFRSF11B
SLC2A10
GJA1
TTN
OXT
PTLS
VCL
WT1
ITGB3
ATORS
FDH
MOWS
SQT1
RAMSVPS
HTX6
CMH9
SRTD15
THPH9
NPPC
CAV1
LAMA4
GHRHR
PTGIS
NAT8
GUCY1B2
MIR145
SLC16A9
KAT6A
CD36
RETN
ALDH2
PDA1
FGG
FABP4
INS
HSD17B7
MTTQ
IRS1
AOS5
HLHS1

FLT4
KLF5
ALGS1
DRD3
GTF2I
TMEM88
PAFAD
AGTR2
ATE1
PAPPA
PITA4
MCOPS1
MKKS
B12QTL1
CHDS6
OI13
ARCL2C
ARHI1
GAS6
CD47
KCNMA1
HOXA1
ESS2
TLL1
USP16
OTUD6B
IFT140
SEPT8
PKD2
UCN3
ADRB2
CETP
MYH7
KCNJ2
F2
EVC2
UTS2
PTGIR
SGBS1
JDSCD
CPHD1
ATFB13
KCNJ4
IL6R
S1PR1
MIR143
PTGER1
PIAS4
CPS1
CHRNA3
NPY
SDHA

SST
TAC4
EDSVASC
COL4A1
SKI
SCARB1
GLA
PNPLA2

TwHF and CVD Common targets
CYP19A1
G6PD
AR
HSD11B1
VDR
GABRA2
GABRB2
GABRG2
FABP4
PPARA
PPARD
FABP2
FFAR1
PLG
PRKCA
ALOX5AP
SHH
NR1H2
ESR1
ESR2
CA4
TRPV1
JAK2
AGTR1
HMGCR
GLRA1
KCNH2
KDR
HTR2B
MAPK14
F2R
PLA2G7
SLC6A2
APP
MAPK13
NR1H3
CYP17A1
CYP2C19
SLC6A4
BCHE
CHRM2
PTGER1
SQLE
NOS2
NR3C1
PPARG
DRD2
PTGIR
TERT
PTPN11

SCD
CCR5
PRKCB
ALOX5
PTGS2
NR3C2
PTGS1
SLC6A3
PRKCH
IL6
MMP3
MMP1
MMP2
EDNRB
EDNRA
ITGB1
MME
ACE
TNF
ITGB3
TYMS
EPHB4
EPHB6
THRA
MMP9
XDH
CA2
CA7
CA12
CETP
MAPT
INSR
MYLK
SYK
PIK3CG
PIK3CA
EGFR
F2
MPO
CA1
CA3
CA6
CA14
CA9
CSNK2A1
CA5A
KIT
TNNT2
TNNI3
ECE1
TP53
TBXA2R

FLT1
MTOR
HTR2A
RBP4
TGFBR1
ABCB1
CTSD
REN
CASP8
ADAM17
ADAM10
CCND1
DRD3
STAT3
ADRA1A
HTR2C
GBA
ADK
NTRK1
BRAF
ADRB1
VHL
LIPG
GABRA5
CASR
MAPK1
PSEN2
PSEN1
SCARB1
BCL2
AGTR2
FGFR1
PDGFRB
PTPN22
PLA2G2A
NLRP3
ABCC9
F9
BDKRB2
TSHR
LDLR
GABRB3
GABRA3
GABRA1
HLA-DRB1
LIPA
TRPV4
KCNQ1
HSD17B7
CYP11B2
FLT4
LRRK2

ADRA1D
IL1B
LPL
ELANE
ALDH2
CA5B
FOLH1
HRAS
ALB
SERPINE1
NRP1
KLK1
RET
ADRA2B
CAPNS1
ADRA1B
AKT1
ADRB2
CX3CR1
LIPC
NR1I2
CHRNA3
CHRM1
S1PR1
