# Supplementary material for: Exploring the Pharmacological Mechanisms of Tripterygium wilfordii Hook F against Cardiovascular Disease Using Network Pharmacology and Molecular Docking
Source: Biomed Res Int. 2021 Aug 14;2021:5575621. doi: 10.1155/2021/5575621 (PMC8382521; doi:10.1155/2021/5575621)
Supplement: Supplementary Materials — Known targets of TwHF and known therapeutic targets acting on CVD were shown in Supplementary Table 1. Known protein interactions of TwHF against CVD (combined score ≥ 0.9) were shown in Supplementary Table 2. [file 5575621.f1.zip › Supplementary Table 2.pdf]

#node1	node2	node1_st	node2_st	node1_ex	node2_ex	neighbor	gene_fus	phylogen	homology
ACE	REN	1847260	1846232	9606.ENS	9606.ENS	0	0	0	0
ADAM17	EGFR	1849242	1846445	9606.ENS	9606.ENS	0	0	0	0
ADAM17	ADAM10	1849242	1844789	9606.ENS	9606.ENS	0	0	0	0.721
ADAM17	PRKCB	1849242	1848723	9606.ENS	9606.ENS	0	0	0	0
ADRA1A	ADRA1B	1857247	1848870	9606.ENS	9606.ENS	0	0	0	0.946
ADRA1A	ADRA1D	1857247	1857034	9606.ENS	9606.ENS	0	0	0	0.939
ADRA1A	AGTR1	1857247	1846323	9606.ENS	9606.ENS	0	0	0	0.65
ADRA1A	CHRM1	1857247	1848849	9606.ENS	9606.ENS	0	0	0	0.677
ADRA1A	HTR2A	1857247	1856916	9606.ENS	9606.ENS	0	0	0	0.728
ADRA1A	HTR2B	1857247	1844597	9606.ENS	9606.ENS	0	0	0	0.703
ADRA1A	EDNRB	1857247	1856642	9606.ENS	9606.ENS	0	0	0	0.628
ADRA1A	PIK3CA	1857247	1845446	9606.ENS	9606.ENS	0	0	0	0
ADRA1A	EDNRA	1857247	1849748	9606.ENS	9606.ENS	0	0	0	0.624
ADRA1A	BDKRB2	1857247	1848988	9606.ENS	9606.ENS	0	0	0	0.643
ADRA1A	F2R	1857247	1850333	9606.ENS	9606.ENS	0	0	0	0.623
ADRA1A	PTGER1	1857247	1847360	9606.ENS	9606.ENS	0	0	0	0
ADRA1A	APP	1857247	1846936	9606.ENS	9606.ENS	0	0	0	0
ADRA1A	FFAR1	1857247	1843825	9606.ENS	9606.ENS	0	0	0	0
ADRA1B	ADRB2	1848870	1848724	9606.ENS	9606.ENS	0	0	0	0.818
ADRA1B	APP	1848870	1846936	9606.ENS	9606.ENS	0	0	0	0
ADRA1B	AGTR1	1848870	1846323	9606.ENS	9606.ENS	0	0	0	0.636
ADRA1B	HTR2B	1848870	1844597	9606.ENS	9606.ENS	0	0	0	0.72
ADRA1B	CCR5	1848870	1847342	9606.ENS	9606.ENS	0	0	0	0.627
ADRA1B	CHRM1	1848870	1848849	9606.ENS	9606.ENS	0	0	0	0.671
ADRA1B	PIK3CA	1848870	1845446	9606.ENS	9606.ENS	0	0	0	0
ADRA1B	HTR2C	1848870	1846478	9606.ENS	9606.ENS	0	0	0	0.748
ADRA1B	PTGER1	1848870	1847360	9606.ENS	9606.ENS	0	0	0	0
ADRA1B	FFAR1	1848870	1843825	9606.ENS	9606.ENS	0	0	0	0
ADRA1D	CHRM1	1857034	1848849	9606.ENS	9606.ENS	0	0	0	0.683
ADRA1D	HTR2B	1857034	1844597	9606.ENS	9606.ENS	0	0	0	0.701
ADRA1D	AGTR1	1857034	1846323	9606.ENS	9606.ENS	0	0	0	0.643
ADRA1D	HTR2A	1857034	1856916	9606.ENS	9606.ENS	0	0	0	0.724
ADRA1D	EDNRA	1857034	1849748	9606.ENS	9606.ENS	0	0	0	0
ADRA1D	ADRA1B	1857034	1848870	9606.ENS	9606.ENS	0	0	0	0.935
ADRA1D	F2R	1857034	1850333	9606.ENS	9606.ENS	0	0	0	0
ADRA1D	EDNRB	1857034	1856642	9606.ENS	9606.ENS	0	0	0	0.622
ADRA1D	PIK3CA	1857034	1845446	9606.ENS	9606.ENS	0	0	0	0
ADRA1D	APP	1857034	1846936	9606.ENS	9606.ENS	0	0	0	0
ADRA1D	BDKRB2	1857034	1848988	9606.ENS	9606.ENS	0	0	0	0.63
ADRA1D	FFAR1	1857034	1843825	9606.ENS	9606.ENS	0	0	0	0
ADRA1D	PTGER1	1857034	1847360	9606.ENS	9606.ENS	0	0	0	0
ADRA2B	CHRM2	1859258	1850196	9606.ENS	9606.ENS	0	0	0	0.705
ADRA2B	DRD2	1859258	1854237	9606.ENS	9606.ENS	0	0	0	0.81
ADRA2B	DRD3	1859258	1857726	9606.ENS	9606.ENS	0	0	0	0.727
ADRA2B	S1PR1	1859258	1848727	9606.ENS	9606.ENS	0	0	0	0.626
ADRA2B	APP	1859258	1846936	9606.ENS	9606.ENS	0	0	0	0
ADRA2B	BDKRB2	1859258	1848988	9606.ENS	9606.ENS	0	0	0	0
ADRA2B	CCR5	1859258	1847342	9606.ENS	9606.ENS	0	0	0	0.631
ADRB1	ADRB2	1854995	1848724	9606.ENS	9606.ENS	0	0	0	0.946
ADRB1	TSHR	1854995	1847934	9606.ENS	9606.ENS	0	0	0	0
ADRB1	PTGIR	1854995	1847278	9606.ENS	9606.ENS	0	0	0	0
ADRB2	PTGER1	1848724	1847360	9606.ENS	9606.ENS	0	0	0	0
ADRB2	TSHR	1848724	1847934	9606.ENS	9606.ENS	0	0	0	0

ADRB2	VHL	1848724	1844441	9606.ENSI	9606.ENSI	0	0	0	0
ADRB2	CCR5	1848724	1847342	9606.ENSI	9606.ENSI	0	0	0	0.636
ADRB2	HTR2C	1848724	1846478	9606.ENSI	9606.ENSI	0	0	0	0.726
ADRB2	PTGIR	1848724	1847278	9606.ENSI	9606.ENSI	0	0	0	0
AGTR1	HTR2B	1846323	1844597	9606.ENSI	9606.ENSI	0	0	0	0.649
AGTR1	PIK3CA	1846323	1845446	9606.ENSI	9606.ENSI	0	0	0	0
AGTR1	FFAR1	1846323	1843825	9606.ENSI	9606.ENSI	0	0	0	0
AKT1	PIK3CA	1846136	1845446	9606.ENSI	9606.ENSI	0	0	0	0
AKT1	ESR1	1846136	1842435	9606.ENSI	9606.ENSI	0	0	0	0
AKT1	RAF1	1846136	1844086	9606.ENSI	9606.ENSI	0	0	0	0.635
AKT1	RPS6KB1	1846136	1843035	9606.ENSI	9606.ENSI	0	0	0.432	0.87
AKT1	CCND1	1846136	1843117	9606.ENSI	9606.ENSI	0	0	0	0
AKT1	TP53	1846136	1846083	9606.ENSI	9606.ENSI	0	0	0	0
AKT1	STAT3	1846136	1845584	9606.ENSI	9606.ENSI	0	0	0	0
AKT1	ITGB3	1846136	1845040	9606.ENSI	9606.ENSI	0	0	0	0
AKT1	MAPK14	1846136	1843222	9606.ENSI	9606.ENSI	0	0	0	0.67
AKT1	IL6	1846136	1844631	9606.ENSI	9606.ENSI	0	0	0	0
AKT1	MMP2	1846136	1842719	9606.ENSI	9606.ENSI	0	0	0	0
AKT1	PDGFRB	1846136	1844996	9606.ENSI	9606.ENSI	0	0	0	0.617
AKT1	SERPINE1	1846136	1842959	9606.ENSI	9606.ENSI	0	0	0	0
ALB	SERPINE1	1847637	1842959	9606.ENSI	9606.ENSI	0	0	0	0
ALB	TP53	1847637	1846083	9606.ENSI	9606.ENSI	0	0	0	0
ALB	APP	1847637	1846936	9606.ENSI	9606.ENSI	0	0	0	0
ALB	KDR	1847637	1845437	9606.ENSI	9606.ENSI	0	0	0	0
ALB	CTSD	1847637	1843439	9606.ENSI	9606.ENSI	0	0	0	0
ALOX5	PTGS2	1856107	1854602	9606.ENSI	9606.ENSI	0	0	0	0
ALOX5AP	ALOX5	1857231	1856107	9606.ENSI	9606.ENSI	0	0	0	0
APP	ADAM10	1846936	1844789	9606.ENSI	9606.ENSI	0	0	0	0
APP	MAPK1	1846936	1842512	9606.ENSI	9606.ENSI	0	0	0	0
APP	F9	1846936	1842692	9606.ENSI	9606.ENSI	0	0	0	0
APP	SERPINE1	1846936	1842959	9606.ENSI	9606.ENSI	0	0	0	0
APP	AGTR1	1846936	1846323	9606.ENSI	9606.ENSI	0	0	0	0
APP	PIK3CA	1846936	1845446	9606.ENSI	9606.ENSI	0	0	0	0
APP	HTR2B	1846936	1844597	9606.ENSI	9606.ENSI	0	0	0	0
APP	FFAR1	1846936	1843825	9606.ENSI	9606.ENSI	0	0	0	0
AR	CCND1	1856164	1843117	9606.ENSI	9606.ENSI	0	0	0	0
AR	EGFR	1856164	1846445	9606.ENSI	9606.ENSI	0	0	0	0
AR	AKT1	1856164	1846136	9606.ENSI	9606.ENSI	0	0	0	0
AR	STAT3	1856164	1845584	9606.ENSI	9606.ENSI	0	0	0	0
AR	CDK6	1856164	1845799	9606.ENSI	9606.ENSI	0	0	0	0
AR	TP53	1856164	1846083	9606.ENSI	9606.ENSI	0	0	0	0
AR	ESR1	1856164	1842435	9606.ENSI	9606.ENSI	0	0	0	0.692
AR	CASP8	1856164	1853692	9606.ENSI	9606.ENSI	0	0	0	0
AR	NR3C1	1856164	1843294	9606.ENSI	9606.ENSI	0	0	0	0.825
AR	HRAS	1856164	1849235	9606.ENSI	9606.ENSI	0	0	0	0
AR	PIK3CA	1856164	1845446	9606.ENSI	9606.ENSI	0	0	0	0
AR	MAPK14	1856164	1843222	9606.ENSI	9606.ENSI	0	0	0	0
AR	IL6	1856164	1844631	9606.ENSI	9606.ENSI	0	0	0	0
AR	ESR2	1856164	1852756	9606.ENSI	9606.ENSI	0	0	0	0.707
AR	PPARG	1856164	1847090	9606.ENSI	9606.ENSI	0	0	0	0.626
AR	PPARA	1856164	1845205	9606.ENSI	9606.ENSI	0	0	0	0.638
AR	THRA	1856164	1845577	9606.ENSI	9606.ENSI	0	0	0	0.635
AR	PPARD	1856164	1849364	9606.ENSI	9606.ENSI	0	0	0	0.629
AR	NR1H2	1856164	1844246	9606.ENSI	9606.ENSI	0	0	0	0.641

BCL2	RAF1	1851168	1844086	9606.ENSI	9606.ENSI	0	0	0	0
BCL2	TP53	1851168	1846083	9606.ENSI	9606.ENSI	0	0	0	0
BCL2	AKT1	1851168	1846136	9606.ENSI	9606.ENSI	0	0	0	0
BCL2	MAPK1	1851168	1842512	9606.ENSI	9606.ENSI	0	0	0	0
BCL2	IRS1	1851168	1848682	9606.ENSI	9606.ENSI	0	0	0	0
BCL2	CCND1	1851168	1843117	9606.ENSI	9606.ENSI	0	0	0	0
BCL2	HRAS	1851168	1849235	9606.ENSI	9606.ENSI	0	0	0	0
BCL2	MAPK14	1851168	1843222	9606.ENSI	9606.ENSI	0	0	0	0
BCL2	PSEN1	1851168	1850825	9606.ENSI	9606.ENSI	0	0	0	0
BCL2	ALB	1851168	1847637	9606.ENSI	9606.ENSI	0	0	0	0
BCL2	IL6	1851168	1844631	9606.ENSI	9606.ENSI	0	0	0	0
BCL2	MMP2	1851168	1842719	9606.ENSI	9606.ENSI	0	0	0	0
BDKRB2	AGTR1	1848988	1846323	9606.ENSI	9606.ENSI	0	0	0	0.808
BDKRB2	CHRM1	1848988	1848849	9606.ENSI	9606.ENSI	0	0	0	0
BDKRB2	PTGER1	1848988	1847360	9606.ENSI	9606.ENSI	0	0	0	0
BDKRB2	TP53	1848988	1846083	9606.ENSI	9606.ENSI	0	0	0	0
BDKRB2	CCR5	1848988	1847342	9606.ENSI	9606.ENSI	0	0	0	0.735
BDKRB2	PIK3CA	1848988	1845446	9606.ENSI	9606.ENSI	0	0	0	0
BDKRB2	FFAR1	1848988	1843825	9606.ENSI	9606.ENSI	0	0	0	0
BDKRB2	APP	1848988	1846936	9606.ENSI	9606.ENSI	0	0	0	0
BDKRB2	HTR2B	1848988	1844597	9606.ENSI	9606.ENSI	0	0	0	0.624
BDKRB2	S1PR1	1848988	1848727	9606.ENSI	9606.ENSI	0	0	0	0
BDKRB2	ADRA1B	1848988	1848870	9606.ENSI	9606.ENSI	0	0	0	0.645
BRAF	RAF1	1847131	1844086	9606.ENSI	9606.ENSI	0	0	0	0.958
BRAF	MAPK1	1847131	1842512	9606.ENSI	9606.ENSI	0	0	0	0.637
BRAF	AKT1	1847131	1846136	9606.ENSI	9606.ENSI	0	0	0	0.637
CAPNS1	MAPK1	1843820	1842512	9606.ENSI	9606.ENSI	0	0	0	0
CASP8	BCL2	1853692	1851168	9606.ENSI	9606.ENSI	0	0	0	0
CASP8	MAPK1	1853692	1842512	9606.ENSI	9606.ENSI	0	0	0	0
CASP8	APP	1853692	1846936	9606.ENSI	9606.ENSI	0	0	0	0
CASP8	TP53	1853692	1846083	9606.ENSI	9606.ENSI	0	0	0	0
CASP8	ITGB3	1853692	1845040	9606.ENSI	9606.ENSI	0	0	0	0
CASR	APP	1861464	1846936	9606.ENSI	9606.ENSI	0	0	0	0
CASR	EDNRB	1861464	1856642	9606.ENSI	9606.ENSI	0	0	0	0
CASR	AGTR1	1861464	1846323	9606.ENSI	9606.ENSI	0	0	0	0
CASR	CCR5	1861464	1847342	9606.ENSI	9606.ENSI	0	0	0	0
CASR	F2R	1861464	1850333	9606.ENSI	9606.ENSI	0	0	0	0
CASR	DRD2	1861464	1854237	9606.ENSI	9606.ENSI	0	0	0	0
CASR	CHRM2	1861464	1850196	9606.ENSI	9606.ENSI	0	0	0	0
CASR	CHRM1	1861464	1848849	9606.ENSI	9606.ENSI	0	0	0	0
CASR	EDNRA	1861464	1849748	9606.ENSI	9606.ENSI	0	0	0	0
CASR	TBXA2R	1861464	1859713	9606.ENSI	9606.ENSI	0	0	0	0
CASR	ADRA1B	1861464	1848870	9606.ENSI	9606.ENSI	0	0	0	0
CASR	FFAR1	1861464	1843825	9606.ENSI	9606.ENSI	0	0	0	0
CASR	PIK3CA	1861464	1845446	9606.ENSI	9606.ENSI	0	0	0	0
CASR	HTR2B	1861464	1844597	9606.ENSI	9606.ENSI	0	0	0	0
CASR	ADRA1D	1861464	1857034	9606.ENSI	9606.ENSI	0	0	0	0
CASR	HTR2A	1861464	1856916	9606.ENSI	9606.ENSI	0	0	0	0
CASR	ADRA1A	1861464	1857247	9606.ENSI	9606.ENSI	0	0	0	0
CASR	DRD3	1861464	1857726	9606.ENSI	9606.ENSI	0	0	0	0
CASR	PTGER1	1861464	1847360	9606.ENSI	9606.ENSI	0	0	0	0
CASR	ADRA2B	1861464	1859258	9606.ENSI	9606.ENSI	0	0	0	0
CASR	S1PR1	1861464	1848727	9606.ENSI	9606.ENSI	0	0	0	0
CASR	BDKRB2	1861464	1848988	9606.ENSI	9606.ENSI	0	0	0	0

CCND1	ESR1	1843117	1842435	9606.ENSI	9606.ENSI	0	0	0	0
CCND1	MAPK1	1843117	1842512	9606.ENSI	9606.ENSI	0	0	0	0
CCR5	STAT3	1847342	1845584	9606.ENSI	9606.ENSI	0	0	0	0
CCR5	APP	1847342	1846936	9606.ENSI	9606.ENSI	0	0	0	0
CCR5	HTR2C	1847342	1846478	9606.ENSI	9606.ENSI	0	0	0	0
CDK4	CCND1	1844551	1843117	9606.ENSI	9606.ENSI	0	0	0	0
CDK4	FABP4	1844551	1844407	9606.ENSI	9606.ENSI	0	0	0	0
CDK6	CCND1	1845799	1843117	9606.ENSI	9606.ENSI	0	0	0	0
CDK6	CDK4	1845799	1844551	9606.ENSI	9606.ENSI	0	0	0.526	0.977
CHRM1	APP	1848849	1846936	9606.ENSI	9606.ENSI	0	0	0	0
CHRM1	ADRB2	1848849	1848724	9606.ENSI	9606.ENSI	0	0	0	0.7
CHRM1	FFAR1	1848849	1843825	9606.ENSI	9606.ENSI	0	0	0	0
CHRM1	PIK3CA	1848849	1845446	9606.ENSI	9606.ENSI	0	0	0	0
CHRM1	AGTR1	1848849	1846323	9606.ENSI	9606.ENSI	0	0	0	0.636
CHRM1	HTR2B	1848849	1844597	9606.ENSI	9606.ENSI	0	0	0	0.645
CHRM1	HTR2C	1848849	1846478	9606.ENSI	9606.ENSI	0	0	0	0.66
CHRM1	CCR5	1848849	1847342	9606.ENSI	9606.ENSI	0	0	0	0
CHRM1	PTGER1	1848849	1847360	9606.ENSI	9606.ENSI	0	0	0	0
CHRM1	ITGB1	1848849	1848520	9606.ENSI	9606.ENSI	0	0	0	0
CHRM2	APP	1850196	1846936	9606.ENSI	9606.ENSI	0	0	0	0
CHRM2	S1PR1	1850196	1848727	9606.ENSI	9606.ENSI	0	0	0	0.624
CHRM2	CCR5	1850196	1847342	9606.ENSI	9606.ENSI	0	0	0	0
CHRM2	BDKRB2	1850196	1848988	9606.ENSI	9606.ENSI	0	0	0	0
CTSD	ESR1	1843439	1842435	9606.ENSI	9606.ENSI	0	0	0	0
CYP11B2	HSD11B1	1850775	1844918	9606.ENSI	9606.ENSI	0	0	0	0
CYP11B2	CYP19A1	1850775	1844790	9606.ENSI	9606.ENSI	0	0	0	0.623
CYP17A1	ESR1	1855129	1842435	9606.ENSI	9606.ENSI	0	0	0	0
CYP17A1	CYP19A1	1855129	1844790	9606.ENSI	9606.ENSI	0	0	0.526	0.63
CYP17A1	CYP11B2	1855129	1850775	9606.ENSI	9606.ENSI	0	0	0	0.626
CYP17A1	ESR2	1855129	1852756	9606.ENSI	9606.ENSI	0	0	0	0
CYP19A1	HSD17B7	1844790	1844300	9606.ENSI	9606.ENSI	0	0	0	0
CYP19A1	ESR1	1844790	1842435	9606.ENSI	9606.ENSI	0	0	0	0
CYP2C19	PTGS2	1855462	1854602	9606.ENSI	9606.ENSI	0	0	0	0
CYP2C19	NR1I2	1855462	1851892	9606.ENSI	9606.ENSI	0	0	0	0
CYP2C19	PTGS1	1855462	1854170	9606.ENSI	9606.ENSI	0	0	0	0
DRD2	SLC6A3	1854237	1846148	9606.ENSI	9606.ENSI	0	0	0	0
DRD2	CHRM2	1854237	1850196	9606.ENSI	9606.ENSI	0	0	0	0.717
DRD2	CCR5	1854237	1847342	9606.ENSI	9606.ENSI	0	0	0	0
DRD2	APP	1854237	1846936	9606.ENSI	9606.ENSI	0	0	0	0
DRD2	BDKRB2	1854237	1848988	9606.ENSI	9606.ENSI	0	0	0	0.625
DRD2	S1PR1	1854237	1848727	9606.ENSI	9606.ENSI	0	0	0	0.644
DRD3	CHRM2	1857726	1850196	9606.ENSI	9606.ENSI	0	0	0	0.71
DRD3	S1PR1	1857726	1848727	9606.ENSI	9606.ENSI	0	0	0	0.632
DRD3	DRD2	1857726	1854237	9606.ENSI	9606.ENSI	0	0	0	0.947
DRD3	APP	1857726	1846936	9606.ENSI	9606.ENSI	0	0	0	0
DRD3	BDKRB2	1857726	1848988	9606.ENSI	9606.ENSI	0	0	0	0
DRD3	CCR5	1857726	1847342	9606.ENSI	9606.ENSI	0	0	0	0
EDNRA	AKT1	1849748	1846136	9606.ENSI	9606.ENSI	0	0	0	0
EDNRA	EGFR	1849748	1846445	9606.ENSI	9606.ENSI	0	0	0	0
EDNRA	APP	1849748	1846936	9606.ENSI	9606.ENSI	0	0	0	0
EDNRA	AGTR1	1849748	1846323	9606.ENSI	9606.ENSI	0	0	0	0.653
EDNRA	PTGER1	1849748	1847360	9606.ENSI	9606.ENSI	0	0	0	0
EDNRA	BDKRB2	1849748	1848988	9606.ENSI	9606.ENSI	0	0	0	0.627
EDNRA	HTR2B	1849748	1844597	9606.ENSI	9606.ENSI	0	0	0	0.62

EDNRA	CHRM1	1849748	1848849	9606.ENSI	9606.ENSI	0	0	0	0
EDNRA	ADRA1B	1849748	1848870	9606.ENSI	9606.ENSI	0	0	0	0.625
EDNRA	FFAR1	1849748	1843825	9606.ENSI	9606.ENSI	0	0	0	0
EDNRA	PIK3CA	1849748	1845446	9606.ENSI	9606.ENSI	0	0	0	0
EDNRB	MAPK14	1856642	1843222	9606.ENSI	9606.ENSI	0	0	0	0
EDNRB	AGTR1	1856642	1846323	9606.ENSI	9606.ENSI	0	0	0	0.662
EDNRB	HTR2B	1856642	1844597	9606.ENSI	9606.ENSI	0	0	0	0.621
EDNRB	APP	1856642	1846936	9606.ENSI	9606.ENSI	0	0	0	0
EDNRB	F2R	1856642	1850333	9606.ENSI	9606.ENSI	0	0	0	0.626
EDNRB	BDKRB2	1856642	1848988	9606.ENSI	9606.ENSI	0	0	0	0.646
EDNRB	PTGER1	1856642	1847360	9606.ENSI	9606.ENSI	0	0	0	0
EDNRB	EDNRA	1856642	1849748	9606.ENSI	9606.ENSI	0	0	0	0.957
EDNRB	ADRA1B	1856642	1848870	9606.ENSI	9606.ENSI	0	0	0	0.624
EDNRB	CHRM1	1856642	1848849	9606.ENSI	9606.ENSI	0	0	0	0
EDNRB	PIK3CA	1856642	1845446	9606.ENSI	9606.ENSI	0	0	0	0
EDNRB	FFAR1	1856642	1843825	9606.ENSI	9606.ENSI	0	0	0	0
EGFR	STAT3	1846445	1845584	9606.ENSI	9606.ENSI	0	0	0	0
EGFR	TP53	1846445	1846083	9606.ENSI	9606.ENSI	0	0	0	0
EGFR	ESR1	1846445	1842435	9606.ENSI	9606.ENSI	0	0	0	0
EGFR	PIK3CA	1846445	1845446	9606.ENSI	9606.ENSI	0	0	0	0
EGFR	MAPK1	1846445	1842512	9606.ENSI	9606.ENSI	0	0	0	0.632
EGFR	CCND1	1846445	1843117	9606.ENSI	9606.ENSI	0	0	0	0
EGFR	ADAM10	1846445	1844789	9606.ENSI	9606.ENSI	0	0	0	0
EGFR	IL6	1846445	1844631	9606.ENSI	9606.ENSI	0	0	0	0
EGFR	MAPK14	1846445	1843222	9606.ENSI	9606.ENSI	0	0	0	0.632
EGFR	PPARA	1846445	1845205	9606.ENSI	9606.ENSI	0	0	0	0
EIF4EBP1	AKT1	1852358	1846136	9606.ENSI	9606.ENSI	0	0	0	0
EIF4EBP1	RPTOR	1852358	1848942	9606.ENSI	9606.ENSI	0	0	0	0
EIF4EBP1	LRRK2	1852358	1848005	9606.ENSI	9606.ENSI	0	0	0	0
EIF4EBP1	RPS6KB1	1852358	1843035	9606.ENSI	9606.ENSI	0	0	0	0
EIF4EBP1	MAPK1	1852358	1842512	9606.ENSI	9606.ENSI	0	0	0	0
EIF4EBP1	MAPK14	1852358	1843222	9606.ENSI	9606.ENSI	0	0	0	0
EIF4EBP1	CCND1	1852358	1843117	9606.ENSI	9606.ENSI	0	0	0	0
EIF4EBP1	PIK3CA	1852358	1845446	9606.ENSI	9606.ENSI	0	0	0	0
ELANE	MMP2	1845377	1842719	9606.ENSI	9606.ENSI	0	0	0	0
EPHB4	PIK3CA	1853646	1845446	9606.ENSI	9606.ENSI	0	0	0	0
ESR2	ESR1	1852756	1842435	9606.ENSI	9606.ENSI	0	0	0	0.935
ESR2	PPARG	1852756	1847090	9606.ENSI	9606.ENSI	0	0	0	0.636
ESR2	MAPK1	1852756	1842512	9606.ENSI	9606.ENSI	0	0	0	0
ESR2	THRA	1852756	1845577	9606.ENSI	9606.ENSI	0	0	0	0.663
ESR2	PPARA	1852756	1845205	9606.ENSI	9606.ENSI	0	0	0	0.642
ESR2	PPARD	1852756	1849364	9606.ENSI	9606.ENSI	0	0	0	0.637
ESR2	NR1H2	1852756	1844246	9606.ENSI	9606.ENSI	0	0	0	0.684
F2	SERPINE1	1849080	1842959	9606.ENSI	9606.ENSI	0.083	0	0	0
F2	F9	1849080	1842692	9606.ENSI	9606.ENSI	0	0	0	0.739
F2	MAPK1	1849080	1842512	9606.ENSI	9606.ENSI	0	0	0	0
F2	PPARA	1849080	1845205	9606.ENSI	9606.ENSI	0	0	0	0
F2	RAF1	1849080	1844086	9606.ENSI	9606.ENSI	0	0	0	0
F2	ESR1	1849080	1842435	9606.ENSI	9606.ENSI	0	0	0	0
F2	AKT1	1849080	1846136	9606.ENSI	9606.ENSI	0	0	0	0
F2	CCR5	1849080	1847342	9606.ENSI	9606.ENSI	0	0	0	0
F2	HTR2C	1849080	1846478	9606.ENSI	9606.ENSI	0	0	0	0
F2	EGFR	1849080	1846445	9606.ENSI	9606.ENSI	0	0	0	0
F2	PTGER1	1849080	1847360	9606.ENSI	9606.ENSI	0	0	0	0

F2	ADRB2	1849080	1848724	9606.ENSl	9606.ENSl	0	0	0	0
F2	CHRM1	1849080	1848849	9606.ENSl	9606.ENSl	0	0	0	0
F2	ADRA1B	1849080	1848870	9606.ENSl	9606.ENSl	0	0	0	0
F2R	F2	1850333	1849080	9606.ENSl	9606.ENSl	0	0	0	0
F2R	PLG	1850333	1849130	9606.ENSl	9606.ENSl	0	0	0	0
F2R	EDNRA	1850333	1849748	9606.ENSl	9606.ENSl	0	0	0	0
F2R	BDKRB2	1850333	1848988	9606.ENSl	9606.ENSl	0	0	0	0.657
F2R	PTGER1	1850333	1847360	9606.ENSl	9606.ENSl	0	0	0	0
F2R	CHRM1	1850333	1848849	9606.ENSl	9606.ENSl	0	0	0	0
F2R	MAPK1	1850333	1842512	9606.ENSl	9606.ENSl	0	0	0	0
F2R	AGTR1	1850333	1846323	9606.ENSl	9606.ENSl	0	0	0	0.731
F2R	ADRA1B	1850333	1848870	9606.ENSl	9606.ENSl	0	0	0	0
F2R	HTR2B	1850333	1844597	9606.ENSl	9606.ENSl	0	0	0	0.622
F2R	APP	1850333	1846936	9606.ENSl	9606.ENSl	0	0	0	0
F2R	FFAR1	1850333	1843825	9606.ENSl	9606.ENSl	0	0	0	0.684
F2R	PIK3CA	1850333	1845446	9606.ENSl	9606.ENSl	0	0	0	0
FGFR1	HRAS	1859710	1849235	9606.ENSl	9606.ENSl	0	0	0	0
FGFR1	CCND1	1859710	1843117	9606.ENSl	9606.ENSl	0	0	0	0
FGFR1	PIK3CA	1859710	1845446	9606.ENSl	9606.ENSl	0	0	0	0
FLT1	NRP1	1846791	1845737	9606.ENSl	9606.ENSl	0	0	0	0
FLT1	KDR	1846791	1845437	9606.ENSl	9606.ENSl	0	0	0	0.918
FLT1	PIK3CA	1846791	1845446	9606.ENSl	9606.ENSl	0	0	0	0
FLT1	FLT4	1846791	1845029	9606.ENSl	9606.ENSl	0	0	0	0.898
GABRA2	GABRB2	1853367	1846395	9606.ENSl	9606.ENSl	0	0	0	0.84
GABRA2	GABRB3	1853367	1848038	9606.ENSl	9606.ENSl	0	0	0	0.853
GABRA2	GABRA1	1853367	1842192	9606.ENSl	9606.ENSl	0	0	0	0.979
GABRA2	GABRA5	1853367	1851869	9606.ENSl	9606.ENSl	0	0	0	0.978
GABRA3	GABRB2	1855218	1846395	9606.ENSl	9606.ENSl	0	0	0	0.828
GABRA3	GABRB3	1855218	1848038	9606.ENSl	9606.ENSl	0	0	0	0.846
GABRA3	GABRA2	1855218	1853367	9606.ENSl	9606.ENSl	0	0	0	0.977
GABRA3	GABRA1	1855218	1842192	9606.ENSl	9606.ENSl	0	0	0	0.976
GABRA3	GABRA5	1855218	1851869	9606.ENSl	9606.ENSl	0	0	0	0.976
GABRA5	GABRB2	1851869	1846395	9606.ENSl	9606.ENSl	0	0	0	0.844
GABRA5	GABRB3	1851869	1848038	9606.ENSl	9606.ENSl	0	0	0	0.852
GABRA5	GABRA1	1851869	1842192	9606.ENSl	9606.ENSl	0	0	0	0.976
GABRB2	GABRA1	1846395	1842192	9606.ENSl	9606.ENSl	0	0	0	0.848
GABRB3	GABRA1	1848038	1842192	9606.ENSl	9606.ENSl	0	0	0	0.859
GABRB3	GABRB2	1848038	1846395	9606.ENSl	9606.ENSl	0	0	0	0.982
GABRG2	GABRA1	1860865	1842192	9606.ENSl	9606.ENSl	0	0	0	0.909
GABRG2	GABRB2	1860865	1846395	9606.ENSl	9606.ENSl	0	0	0	0.82
GABRG2	GABRA2	1860865	1853367	9606.ENSl	9606.ENSl	0	0	0	0.901
GABRG2	GABRA3	1860865	1855218	9606.ENSl	9606.ENSl	0	0	0	0.899
GABRG2	GABRA5	1860865	1851869	9606.ENSl	9606.ENSl	0	0	0	0.9
GABRG2	GABRB3	1860865	1848038	9606.ENSl	9606.ENSl	0	0	0	0.851
HLA-DRB1	CTSD	1853944	1843439	9606.ENSl	9606.ENSl	0	0	0	0
HLA-DRB1	PTPN11	1853944	1852389	9606.ENSl	9606.ENSl	0	0	0	0
HMGCR	PPARA	1847097	1845205	9606.ENSl	9606.ENSl	0	0	0	0
HRAS	RAF1	1849235	1844086	9606.ENSl	9606.ENSl	0	0	0	0
HRAS	PIK3CA	1849235	1845446	9606.ENSl	9606.ENSl	0	0	0	0
HRAS	BRAF	1849235	1847131	9606.ENSl	9606.ENSl	0	0	0	0
HRAS	EGFR	1849235	1846445	9606.ENSl	9606.ENSl	0	0	0	0
HRAS	TP53	1849235	1846083	9606.ENSl	9606.ENSl	0	0	0	0
HRAS	INSR	1849235	1848574	9606.ENSl	9606.ENSl	0	0	0	0
HRAS	KIT	1849235	1847112	9606.ENSl	9606.ENSl	0	0	0	0

HRAS	PDGFRB	1849235	1844996	9606.ENSI	9606.ENSI	0	0	0	0
HRAS	MAPK1	1849235	1842512	9606.ENSI	9606.ENSI	0	0	0	0
HRAS	CCND1	1849235	1843117	9606.ENSI	9606.ENSI	0	0	0	0
HRAS	IRS1	1849235	1848682	9606.ENSI	9606.ENSI	0	0	0	0
HRAS	F2	1849235	1849080	9606.ENSI	9606.ENSI	0	0	0	0
HRAS	PRKCB	1849235	1848723	9606.ENSI	9606.ENSI	0	0	0	0
HTR2A	APP	1856916	1846936	9606.ENSI	9606.ENSI	0	0	0	0
HTR2A	F2R	1856916	1850333	9606.ENSI	9606.ENSI	0	0	0	0.62
HTR2A	EDNRB	1856916	1856642	9606.ENSI	9606.ENSI	0	0	0	0.623
HTR2A	HTR2B	1856916	1844597	9606.ENSI	9606.ENSI	0	0	0	0.918
HTR2A	EDNRA	1856916	1849748	9606.ENSI	9606.ENSI	0	0	0	0.624
HTR2A	AGTR1	1856916	1846323	9606.ENSI	9606.ENSI	0	0	0	0.639
HTR2A	CHRM1	1856916	1848849	9606.ENSI	9606.ENSI	0	0	0	0.651
HTR2A	PTGER1	1856916	1847360	9606.ENSI	9606.ENSI	0	0	0	0
HTR2A	ADRA1B	1856916	1848870	9606.ENSI	9606.ENSI	0	0	0	0.747
HTR2A	FFAR1	1856916	1843825	9606.ENSI	9606.ENSI	0	0	0	0
HTR2A	BDKRB2	1856916	1848988	9606.ENSI	9606.ENSI	0	0	0	0.634
HTR2A	PIK3CA	1856916	1845446	9606.ENSI	9606.ENSI	0	0	0	0
HTR2B	FFAR1	1844597	1843825	9606.ENSI	9606.ENSI	0	0	0	0
IL1B	MAPK14	1845335	1843222	9606.ENSI	9606.ENSI	0	0	0	0
IL1B	IL6	1845335	1844631	9606.ENSI	9606.ENSI	0	0	0	0
IL1B	MMP2	1845335	1842719	9606.ENSI	9606.ENSI	0	0	0	0
IL6	SERPINE1	1844631	1842959	9606.ENSI	9606.ENSI	0	0	0	0
IL6	MMP2	1844631	1842719	9606.ENSI	9606.ENSI	0	0	0	0
IL6	ESR1	1844631	1842435	9606.ENSI	9606.ENSI	0	0	0	0
IL6	CCND1	1844631	1843117	9606.ENSI	9606.ENSI	0	0	0	0
IL6	MAPK14	1844631	1843222	9606.ENSI	9606.ENSI	0	0	0	0
INSR	PIK3CA	1848574	1845446	9606.ENSI	9606.ENSI	0	0	0	0
INSR	AKT1	1848574	1846136	9606.ENSI	9606.ENSI	0	0	0	0.62
INSR	RPS6KB1	1848574	1843035	9606.ENSI	9606.ENSI	0	0	0	0.62
IRS1	INSR	1848682	1848574	9606.ENSI	9606.ENSI	0	0	0	0
IRS1	RPS6KB1	1848682	1843035	9606.ENSI	9606.ENSI	0	0	0	0
IRS1	PIK3CA	1848682	1845446	9606.ENSI	9606.ENSI	0	0	0	0
IRS1	AKT1	1848682	1846136	9606.ENSI	9606.ENSI	0	0	0	0
IRS1	MAPK1	1848682	1842512	9606.ENSI	9606.ENSI	0	0	0	0
IRS1	ESR1	1848682	1842435	9606.ENSI	9606.ENSI	0	0	0	0
IRS1	EGFR	1848682	1846445	9606.ENSI	9606.ENSI	0	0	0	0
IRS1	IL6	1848682	1844631	9606.ENSI	9606.ENSI	0	0	0	0
IRS1	STAT3	1848682	1845584	9606.ENSI	9606.ENSI	0	0	0	0
IRS1	ITGB3	1848682	1845040	9606.ENSI	9606.ENSI	0	0	0	0
IRS1	PDGFRB	1848682	1844996	9606.ENSI	9606.ENSI	0	0	0	0
ITGB1	FLT4	1848520	1845029	9606.ENSI	9606.ENSI	0	0	0	0
ITGB1	KDR	1848520	1845437	9606.ENSI	9606.ENSI	0	0	0	0
ITGB1	EGFR	1848520	1846445	9606.ENSI	9606.ENSI	0	0	0	0
ITGB1	PIK3CA	1848520	1845446	9606.ENSI	9606.ENSI	0	0	0	0
ITGB1	RPS6KB1	1848520	1843035	9606.ENSI	9606.ENSI	0	0	0	0
ITGB1	CAPNS1	1848520	1843820	9606.ENSI	9606.ENSI	0	0	0	0
ITGB3	PDGFRB	1845040	1844996	9606.ENSI	9606.ENSI	0	0	0	0
ITGB3	SERPINE1	1845040	1842959	9606.ENSI	9606.ENSI	0	0	0	0
ITGB3	MAPK1	1845040	1842512	9606.ENSI	9606.ENSI	0	0	0	0
JAK2	PTPN11	1857411	1852389	9606.ENSI	9606.ENSI	0	0	0	0
JAK2	STAT3	1857411	1845584	9606.ENSI	9606.ENSI	0	0	0	0
JAK2	IRS1	1857411	1848682	9606.ENSI	9606.ENSI	0	0	0	0
JAK2	IL6	1857411	1844631	9606.ENSI	9606.ENSI	0	0	0	0

JAK2	KIT	1857411	1847112	9606.ENSI	9606.ENSI	0	0	0	0.619
JAK2	CCR5	1857411	1847342	9606.ENSI	9606.ENSI	0	0	0	0
JAK2	PDGFRB	1857411	1844996	9606.ENSI	9606.ENSI	0	0	0	0.616
JAK2	PIK3CA	1857411	1845446	9606.ENSI	9606.ENSI	0	0	0	0
JAK2	AGTR1	1857411	1846323	9606.ENSI	9606.ENSI	0	0	0	0
JAK2	HRAS	1857411	1849235	9606.ENSI	9606.ENSI	0	0	0	0
JAK2	IL1B	1857411	1845335	9606.ENSI	9606.ENSI	0	0	0	0
JAK2	BCL2	1857411	1851168	9606.ENSI	9606.ENSI	0	0	0	0
JAK2	MAPK1	1857411	1842512	9606.ENSI	9606.ENSI	0	0	0	0.653
JAK2	NOS2	1857411	1850906	9606.ENSI	9606.ENSI	0	0	0	0
JAK2	HTR2A	1857411	1856916	9606.ENSI	9606.ENSI	0	0	0	0
JAK2	PIK3CG	1857411	1853801	9606.ENSI	9606.ENSI	0	0	0	0
JAK2	EDNRA	1857411	1849748	9606.ENSI	9606.ENSI	0	0	0	0
JAK2	EGFR	1857411	1846445	9606.ENSI	9606.ENSI	0	0	0	0.63
JAK2	MPO	1857411	1843011	9606.ENSI	9606.ENSI	0	0	0	0
KCNH2	KCNQ1	1845083	1842257	9606.ENSI	9606.ENSI	0	0	0	0
KDR	FLT4	1845437	1845029	9606.ENSI	9606.ENSI	0	0	0	0.92
KDR	ITGB3	1845437	1845040	9606.ENSI	9606.ENSI	0	0	0	0
KDR	IL6	1845437	1844631	9606.ENSI	9606.ENSI	0	0	0	0
KIT	STAT3	1847112	1845584	9606.ENSI	9606.ENSI	0	0	0	0
KIT	PIK3CA	1847112	1845446	9606.ENSI	9606.ENSI	0	0	0	0
KIT	RPS6KB1	1847112	1843035	9606.ENSI	9606.ENSI	0	0	0	0.617
LDLR	LPL	1862854	1849220	9606.ENSI	9606.ENSI	0	0	0	0
LDLR	LIPC	1862854	1848014	9606.ENSI	9606.ENSI	0	0	0	0
LIPC	LIPG	1848014	1844879	9606.ENSI	9606.ENSI	0	0	0	0.923
LPL	PPARA	1849220	1845205	9606.ENSI	9606.ENSI	0	0	0	0
LPL	PPARG	1849220	1847090	9606.ENSI	9606.ENSI	0	0	0	0
LPL	FABP4	1849220	1844407	9606.ENSI	9606.ENSI	0	0	0	0
LPL	NR3C1	1849220	1843294	9606.ENSI	9606.ENSI	0	0	0	0
LPL	LIPC	1849220	1848014	9606.ENSI	9606.ENSI	0	0	0	0.928
LPL	LIPG	1849220	1844879	9606.ENSI	9606.ENSI	0	0	0	0.943
MAPK1	ESR1	1842512	1842435	9606.ENSI	9606.ENSI	0	0	0	0
MAPK14	MAPK1	1843222	1842512	9606.ENSI	9606.ENSI	0	0	0.342	0.939
MAPK14	ESR1	1843222	1842435	9606.ENSI	9606.ENSI	0	0	0	0
MAPK14	MAPK13	1843222	1842465	9606.ENSI	9606.ENSI	0	0	0.525	0.971
MAPK14	CCND1	1843222	1843117	9606.ENSI	9606.ENSI	0	0	0	0
MAPT	AKT1	1852372	1846136	9606.ENSI	9606.ENSI	0	0	0	0
MAPT	APP	1852372	1846936	9606.ENSI	9606.ENSI	0	0	0	0
MAPT	LRRK2	1852372	1848005	9606.ENSI	9606.ENSI	0	0	0	0
MAPT	PSEN1	1852372	1850825	9606.ENSI	9606.ENSI	0	0	0	0
MLST8	RICTOR	1858459	1853522	9606.ENSI	9606.ENSI	0	0	0	0
MLST8	EIF4EBP1	1858459	1852358	9606.ENSI	9606.ENSI	0	0	0	0
MLST8	RPTOR	1858459	1848942	9606.ENSI	9606.ENSI	0	0	0	0
MLST8	MTOR	1858459	1854147	9606.ENSI	9606.ENSI	0	0	0	0
MLST8	RPS6KB1	1858459	1843035	9606.ENSI	9606.ENSI	0	0	0	0
MLST8	AKT1	1858459	1846136	9606.ENSI	9606.ENSI	0	0	0	0
MLST8	IRS1	1858459	1848682	9606.ENSI	9606.ENSI	0	0	0	0
MME	REN	1854017	1846232	9606.ENSI	9606.ENSI	0	0	0	0
MMP1	EDNRA	1850481	1849748	9606.ENSI	9606.ENSI	0	0	0	0
MMP1	IL6	1850481	1844631	9606.ENSI	9606.ENSI	0	0	0	0
MMP1	IL1B	1850481	1845335	9606.ENSI	9606.ENSI	0	0	0	0
MMP1	MMP2	1850481	1842719	9606.ENSI	9606.ENSI	0	0	0	0.798
MMP1	ELANE	1850481	1845377	9606.ENSI	9606.ENSI	0	0	0	0
MMP1	AKT1	1850481	1846136	9606.ENSI	9606.ENSI	0	0	0	0

MMP1	TP53	1850481	1846083	9606.ENSI	9606.ENSI	0	0	0	0
MMP3	IL1B	1848108	1845335	9606.ENSI	9606.ENSI	0	0	0	0
MMP9	PLG	1855661	1849130	9606.ENSI	9606.ENSI	0	0	0	0
MMP9	AKT1	1855661	1846136	9606.ENSI	9606.ENSI	0	0	0	0
MMP9	IL6	1855661	1844631	9606.ENSI	9606.ENSI	0	0	0	0
MMP9	EGFR	1855661	1846445	9606.ENSI	9606.ENSI	0	0	0	0
MMP9	TP53	1855661	1846083	9606.ENSI	9606.ENSI	0	0	0	0
MMP9	BCL2	1855661	1851168	9606.ENSI	9606.ENSI	0	0	0	0
MMP9	IL1B	1855661	1845335	9606.ENSI	9606.ENSI	0	0	0	0
MMP9	ELANE	1855661	1845377	9606.ENSI	9606.ENSI	0	0	0	0
MMP9	MMP1	1855661	1850481	9606.ENSI	9606.ENSI	0	0	0	0.745
MMP9	STAT3	1855661	1845584	9606.ENSI	9606.ENSI	0	0	0	0
MMP9	FLT1	1855661	1846791	9606.ENSI	9606.ENSI	0	0	0	0
MMP9	MMP2	1855661	1842719	9606.ENSI	9606.ENSI	0	0	0	0.94
MMP9	HRAS	1855661	1849235	9606.ENSI	9606.ENSI	0	0	0	0
MTOR	IRS1	1854147	1848682	9606.ENSI	9606.ENSI	0	0	0	0
MTOR	EIF4EBP1	1854147	1852358	9606.ENSI	9606.ENSI	0	0	0	0
MTOR	RPS6KB1	1854147	1843035	9606.ENSI	9606.ENSI	0	0	0	0
MTOR	STAT3	1854147	1845584	9606.ENSI	9606.ENSI	0	0	0	0
MTOR	RICTOR	1854147	1853522	9606.ENSI	9606.ENSI	0	0	0	0
MTOR	AKT1	1854147	1846136	9606.ENSI	9606.ENSI	0	0	0	0
MTOR	RPTOR	1854147	1848942	9606.ENSI	9606.ENSI	0	0	0	0
MTOR	TERT	1854147	1849199	9606.ENSI	9606.ENSI	0	0	0	0
MTOR	TP53	1854147	1846083	9606.ENSI	9606.ENSI	0	0	0	0
MTOR	EGFR	1854147	1846445	9606.ENSI	9606.ENSI	0	0	0	0
MTOR	PIK3CG	1854147	1853801	9606.ENSI	9606.ENSI	0	0	0	0
MTOR	BCL2	1854147	1851168	9606.ENSI	9606.ENSI	0	0	0	0
MTOR	PIK3CA	1854147	1845446	9606.ENSI	9606.ENSI	0	0	0	0.61
MTOR	CCND1	1854147	1843117	9606.ENSI	9606.ENSI	0	0	0	0
MTOR	PDGFRB	1854147	1844996	9606.ENSI	9606.ENSI	0	0	0	0
MTOR	PTPN11	1854147	1852389	9606.ENSI	9606.ENSI	0	0	0	0
MTOR	IL6	1854147	1844631	9606.ENSI	9606.ENSI	0	0	0	0
MTOR	PPARG	1854147	1847090	9606.ENSI	9606.ENSI	0	0	0	0
MTOR	FLT4	1854147	1845029	9606.ENSI	9606.ENSI	0	0	0	0
MTOR	PRKCB	1854147	1848723	9606.ENSI	9606.ENSI	0	0	0	0
MYLK	ITGB1	1853981	1848520	9606.ENSI	9606.ENSI	0	0	0	0
MYLK	MAPK1	1853981	1842512	9606.ENSI	9606.ENSI	0	0	0	0.636
NLRP3	IL1B	1851990	1845335	9606.ENSI	9606.ENSI	0	0	0	0
NLRP3	APP	1851990	1846936	9606.ENSI	9606.ENSI	0	0	0	0
NOS2	MAPK14	1850906	1843222	9606.ENSI	9606.ENSI	0	0	0	0
NOS2	PPARA	1850906	1845205	9606.ENSI	9606.ENSI	0	0	0	0
NR1H2	ESR1	1844246	1842435	9606.ENSI	9606.ENSI	0	0	0	0.689
NR1H3	PPARA	1859317	1845205	9606.ENSI	9606.ENSI	0	0	0	0.744
NR1H3	LPL	1859317	1849220	9606.ENSI	9606.ENSI	0	0	0	0
NR1H3	NR1H2	1859317	1844246	9606.ENSI	9606.ENSI	0	0	0	0.969
NR1I2	ABCB1	1851892	1845794	9606.ENSI	9606.ENSI	0	0	0	0
NR3C1	SERPINE1	1843294	1842959	9606.ENSI	9606.ENSI	0	0	0	0
NR3C1	ESR1	1843294	1842435	9606.ENSI	9606.ENSI	0	0	0	0.695
NRP1	KDR	1845737	1845437	9606.ENSI	9606.ENSI	0	0	0	0
NTRK1	SHC1	1861874	1860266	9606.ENSI	9606.ENSI	0	0	0	0
NTRK1	STAT3	1861874	1845584	9606.ENSI	9606.ENSI	0	0	0	0
NTRK1	IRS1	1861874	1848682	9606.ENSI	9606.ENSI	0	0	0	0
NTRK1	PIK3CA	1861874	1845446	9606.ENSI	9606.ENSI	0	0	0	0
NTRK1	BRAF	1861874	1847131	9606.ENSI	9606.ENSI	0	0	0	0.624

PDGFRB	RAF1	1844996	1844086	9606.ENSI	9606.ENSI	0	0	0	0.624
PIK3CA	RPS6KB1	1845446	1843035	9606.ENSI	9606.ENSI	0	0	0	0
PIK3CA	ESR1	1845446	1842435	9606.ENSI	9606.ENSI	0	0	0	0
PIK3CA	PDGFRB	1845446	1844996	9606.ENSI	9606.ENSI	0	0	0	0
PIK3CA	CCND1	1845446	1843117	9606.ENSI	9606.ENSI	0	0	0	0
PIK3CA	KDR	1845446	1845437	9606.ENSI	9606.ENSI	0	0	0	0
PIK3CA	FLT4	1845446	1845029	9606.ENSI	9606.ENSI	0	0	0	0
PIK3CA	RAF1	1845446	1844086	9606.ENSI	9606.ENSI	0	0	0	0
PIK3CA	ITGB3	1845446	1845040	9606.ENSI	9606.ENSI	0	0	0	0
PIK3CA	IL1B	1845446	1845335	9606.ENSI	9606.ENSI	0	0	0	0
PIK3CA	NR3C1	1845446	1843294	9606.ENSI	9606.ENSI	0	0	0	0
PIK3CA	HTR2B	1845446	1844597	9606.ENSI	9606.ENSI	0	0	0	0
PIK3CA	FFAR1	1845446	1843825	9606.ENSI	9606.ENSI	0	0	0	0
PIK3CG	HRAS	1853801	1849235	9606.ENSI	9606.ENSI	0	0	0	0
PIK3CG	AKT1	1853801	1846136	9606.ENSI	9606.ENSI	0	0	0	0
PIK3CG	KIT	1853801	1847112	9606.ENSI	9606.ENSI	0	0	0	0
PIK3CG	PTPN11	1853801	1852389	9606.ENSI	9606.ENSI	0	0	0	0
PIK3CG	IRS1	1853801	1848682	9606.ENSI	9606.ENSI	0	0	0	0
PIK3CG	EGFR	1853801	1846445	9606.ENSI	9606.ENSI	0	0	0	0
PIK3CG	PIK3CA	1853801	1845446	9606.ENSI	9606.ENSI	0	0	0	0.83
PLA2G2A	PTGS2	1856239	1854602	9606.ENSI	9606.ENSI	0	0	0	0
PLA2G2A	PLA2G7	1856239	1846414	9606.ENSI	9606.ENSI	0	0	0	0
PLA2G2A	PTGS1	1856239	1854170	9606.ENSI	9606.ENSI	0	0	0	0
PLA2G2A	CYP2C19	1856239	1855462	9606.ENSI	9606.ENSI	0	0	0	0
PLG	SERPINE1	1849130	1842959	9606.ENSI	9606.ENSI	0.083	0	0	0
PLG	ALB	1849130	1847637	9606.ENSI	9606.ENSI	0	0	0	0
PLG	APP	1849130	1846936	9606.ENSI	9606.ENSI	0	0	0	0
PPARA	MAPK1	1845205	1842512	9606.ENSI	9606.ENSI	0	0	0	0
PPARA	FABP4	1845205	1844407	9606.ENSI	9606.ENSI	0	0	0	0
PPARA	SCARB1	1845205	1844967	9606.ENSI	9606.ENSI	0	0	0	0
PPARA	IL6	1845205	1844631	9606.ENSI	9606.ENSI	0	0	0	0
PPARA	NR1H2	1845205	1844246	9606.ENSI	9606.ENSI	0	0	0	0.652
PPARA	ESR1	1845205	1842435	9606.ENSI	9606.ENSI	0	0	0	0.65
PPARD	NR1H2	1849364	1844246	9606.ENSI	9606.ENSI	0	0	0	0.714
PPARD	ESR1	1849364	1842435	9606.ENSI	9606.ENSI	0	0	0	0.65
PPARD	THRA	1849364	1845577	9606.ENSI	9606.ENSI	0	0	0	0.71
PPARD	PPARG	1849364	1847090	9606.ENSI	9606.ENSI	0	0	0	0.967
PPARD	PPARA	1849364	1845205	9606.ENSI	9606.ENSI	0	0	0	0.971
PPARG	ESR1	1847090	1842435	9606.ENSI	9606.ENSI	0	0	0	0.651
PPARG	FABP4	1847090	1844407	9606.ENSI	9606.ENSI	0	0	0	0
PPARG	CDK4	1847090	1844551	9606.ENSI	9606.ENSI	0	0	0	0
PPARG	HSD11B1	1847090	1844918	9606.ENSI	9606.ENSI	0	0	0	0
PPARG	NR1H2	1847090	1844246	9606.ENSI	9606.ENSI	0	0	0	0.726
PPARG	TP53	1847090	1846083	9606.ENSI	9606.ENSI	0	0	0	0
PPARG	SERPINE1	1847090	1842959	9606.ENSI	9606.ENSI	0	0	0	0
PPARG	THRA	1847090	1845577	9606.ENSI	9606.ENSI	0	0	0	0.687
PPARG	IL1B	1847090	1845335	9606.ENSI	9606.ENSI	0	0	0	0
PPARG	PPARA	1847090	1845205	9606.ENSI	9606.ENSI	0	0	0	0.962
PRKCA	BRAF	1860737	1847131	9606.ENSI	9606.ENSI	0	0	0	0.62
PRKCA	EGFR	1860737	1846445	9606.ENSI	9606.ENSI	0	0	0	0.619
PRKCA	TBXA2R	1860737	1859713	9606.ENSI	9606.ENSI	0	0	0	0
PRKCA	RICTOR	1860737	1853522	9606.ENSI	9606.ENSI	0	0	0	0
PRKCA	MTOR	1860737	1854147	9606.ENSI	9606.ENSI	0	0	0	0
PRKCA	RAF1	1860737	1844086	9606.ENSI	9606.ENSI	0	0	0	0.624

PRKCA	ITGB1	1860737	1848520	9606.ENSI	9606.ENSI	0	0	0	0
PRKCA	KIT	1860737	1847112	9606.ENSI	9606.ENSI	0	0	0	0.613
PRKCA	TERT	1860737	1849199	9606.ENSI	9606.ENSI	0	0	0	0
PRKCA	MLST8	1860737	1858459	9606.ENSI	9606.ENSI	0	0	0	0
PRKCA	MAPK1	1860737	1842512	9606.ENSI	9606.ENSI	0	0	0	0.644
PRKCA	BCL2	1860737	1851168	9606.ENSI	9606.ENSI	0	0	0	0
PRKCA	PIK3CA	1860737	1845446	9606.ENSI	9606.ENSI	0	0	0	0
PRKCA	HRAS	1860737	1849235	9606.ENSI	9606.ENSI	0	0	0	0
PRKCA	RPS6KB1	1860737	1843035	9606.ENSI	9606.ENSI	0	0	0	0.838
PRKCA	JAK2	1860737	1857411	9606.ENSI	9606.ENSI	0	0	0	0.617
PRKCA	RET	1860737	1853238	9606.ENSI	9606.ENSI	0	0	0	0.616
PRKCA	ADAM17	1860737	1849242	9606.ENSI	9606.ENSI	0	0	0	0
PRKCA	PRKCB	1860737	1848723	9606.ENSI	9606.ENSI	0	0	0	0.985
PRKCB	EGFR	1848723	1846445	9606.ENSI	9606.ENSI	0	0	0	0.616
PRKCB	RPS6KB1	1848723	1843035	9606.ENSI	9606.ENSI	0	0	0	0.825
PRKCB	RAF1	1848723	1844086	9606.ENSI	9606.ENSI	0	0	0	0.622
PRKCB	BRAF	1848723	1847131	9606.ENSI	9606.ENSI	0	0	0	0.622
PRKCH	MAPK1	1851111	1842512	9606.ENSI	9606.ENSI	0	0	0	0.632
PRKCH	HRAS	1851111	1849235	9606.ENSI	9606.ENSI	0	0	0	0
PSEN1	APP	1850825	1846936	9606.ENSI	9606.ENSI	0	0	0	0
PSEN2	APP	1854447	1846936	9606.ENSI	9606.ENSI	0	0	0	0
PSEN2	ADAM10	1854447	1844789	9606.ENSI	9606.ENSI	0	0	0	0
PSEN2	ADAM17	1854447	1849242	9606.ENSI	9606.ENSI	0	0	0	0
PSEN2	ESR1	1854447	1842435	9606.ENSI	9606.ENSI	0	0	0	0
PTGER1	APP	1847360	1846936	9606.ENSI	9606.ENSI	0	0	0	0
PTGER1	HTR2B	1847360	1844597	9606.ENSI	9606.ENSI	0	0	0	0
PTGER1	FFAR1	1847360	1843825	9606.ENSI	9606.ENSI	0	0	0	0
PTGER1	HTR2C	1847360	1846478	9606.ENSI	9606.ENSI	0	0	0	0
PTGER1	AGTR1	1847360	1846323	9606.ENSI	9606.ENSI	0	0	0	0
PTGER1	CCR5	1847360	1847342	9606.ENSI	9606.ENSI	0	0	0	0
PTGER1	PIK3CA	1847360	1845446	9606.ENSI	9606.ENSI	0	0	0	0
PTGS1	MPO	1854170	1843011	9606.ENSI	9606.ENSI	0	0	0.33	0
PTGS2	TP53	1854602	1846083	9606.ENSI	9606.ENSI	0	0	0	0
PTGS2	MAPK14	1854602	1843222	9606.ENSI	9606.ENSI	0	0	0	0
PTGS2	MAPK1	1854602	1842512	9606.ENSI	9606.ENSI	0	0	0	0
PTGS2	BCL2	1854602	1851168	9606.ENSI	9606.ENSI	0	0	0	0
PTGS2	AKT1	1854602	1846136	9606.ENSI	9606.ENSI	0	0	0	0
PTGS2	PPARA	1854602	1845205	9606.ENSI	9606.ENSI	0	0	0	0
PTGS2	IL6	1854602	1844631	9606.ENSI	9606.ENSI	0	0	0	0
PTGS2	IL1B	1854602	1845335	9606.ENSI	9606.ENSI	0	0	0	0
PTGS2	NOS2	1854602	1850906	9606.ENSI	9606.ENSI	0	0	0	0
PTPN11	KIT	1852389	1847112	9606.ENSI	9606.ENSI	0	0	0	0
PTPN11	EGFR	1852389	1846445	9606.ENSI	9606.ENSI	0	0	0	0
PTPN11	INSR	1852389	1848574	9606.ENSI	9606.ENSI	0	0	0	0
PTPN11	IRS1	1852389	1848682	9606.ENSI	9606.ENSI	0	0	0	0
PTPN11	PDGFRB	1852389	1844996	9606.ENSI	9606.ENSI	0	0	0	0
PTPN11	KDR	1852389	1845437	9606.ENSI	9606.ENSI	0	0	0	0
PTPN11	FLT1	1852389	1846791	9606.ENSI	9606.ENSI	0	0	0	0
PTPN11	IL6	1852389	1844631	9606.ENSI	9606.ENSI	0	0	0	0
PTPN11	AKT1	1852389	1846136	9606.ENSI	9606.ENSI	0	0	0	0
PTPN11	STAT3	1852389	1845584	9606.ENSI	9606.ENSI	0	0	0	0
PTPN11	HRAS	1852389	1849235	9606.ENSI	9606.ENSI	0	0	0	0
PTPN11	MAPK1	1852389	1842512	9606.ENSI	9606.ENSI	0	0	0	0
PTPN11	PIK3CA	1852389	1845446	9606.ENSI	9606.ENSI	0	0	0	0

PTPN11	TP53	1852389	1846083	9606.ENSI	9606.ENSI	0	0	0	0
PTPN11	MAPK14	1852389	1843222	9606.ENSI	9606.ENSI	0	0	0	0
RAF1	MAPK1	1844086	1842512	9606.ENSI	9606.ENSI	0	0	0	0.639
RAF1	CCND1	1844086	1843117	9606.ENSI	9606.ENSI	0	0	0	0
REN	SERPINE1	1846232	1842959	9606.ENSI	9606.ENSI	0	0	0	0
REN	IL6	1846232	1844631	9606.ENSI	9606.ENSI	0	0	0	0
RET	STAT3	1853238	1845584	9606.ENSI	9606.ENSI	0	0	0	0
RET	HRAS	1853238	1849235	9606.ENSI	9606.ENSI	0	0	0	0
RET	PTPN11	1853238	1852389	9606.ENSI	9606.ENSI	0	0	0	0
RET	PIK3CA	1853238	1845446	9606.ENSI	9606.ENSI	0	0	0	0
RET	IRS1	1853238	1848682	9606.ENSI	9606.ENSI	0	0	0	0
RET	MAPK1	1853238	1842512	9606.ENSI	9606.ENSI	0	0	0	0.628
RICTOR	RPS6KB1	1853522	1843035	9606.ENSI	9606.ENSI	0	0	0	0
RICTOR	AKT1	1853522	1846136	9606.ENSI	9606.ENSI	0	0	0	0
RICTOR	EIF4EBP1	1853522	1852358	9606.ENSI	9606.ENSI	0	0	0	0
RICTOR	RPTOR	1853522	1848942	9606.ENSI	9606.ENSI	0	0	0	0
RPS6KB1	MAPK1	1843035	1842512	9606.ENSI	9606.ENSI	0	0	0	0.686
RPTOR	RPS6KB1	1848942	1843035	9606.ENSI	9606.ENSI	0	0	0	0
RPTOR	IRS1	1848942	1848682	9606.ENSI	9606.ENSI	0	0	0	0
RPTOR	AKT1	1848942	1846136	9606.ENSI	9606.ENSI	0	0	0	0
RPTOR	MAPK1	1848942	1842512	9606.ENSI	9606.ENSI	0	0	0	0
S1PR1	AKT1	1848727	1846136	9606.ENSI	9606.ENSI	0	0	0	0
S1PR1	KDR	1848727	1845437	9606.ENSI	9606.ENSI	0	0	0	0
S1PR1	PDGFRB	1848727	1844996	9606.ENSI	9606.ENSI	0	0	0	0
S1PR1	CCR5	1848727	1847342	9606.ENSI	9606.ENSI	0	0	0	0
S1PR1	PIK3CA	1848727	1845446	9606.ENSI	9606.ENSI	0	0	0	0
S1PR1	MAPK1	1848727	1842512	9606.ENSI	9606.ENSI	0	0	0	0
S1PR1	APP	1848727	1846936	9606.ENSI	9606.ENSI	0	0	0	0
SHC1	JAK2	1860266	1857411	9606.ENSI	9606.ENSI	0	0	0	0
SHC1	KDR	1860266	1845437	9606.ENSI	9606.ENSI	0	0	0	0
SHC1	RET	1860266	1853238	9606.ENSI	9606.ENSI	0	0	0	0
SHC1	INSR	1860266	1848574	9606.ENSI	9606.ENSI	0	0	0	0
SHC1	FLT4	1860266	1845029	9606.ENSI	9606.ENSI	0	0	0	0
SHC1	EGFR	1860266	1846445	9606.ENSI	9606.ENSI	0	0	0	0
SHC1	IRS1	1860266	1848682	9606.ENSI	9606.ENSI	0	0	0	0
SHC1	FLT1	1860266	1846791	9606.ENSI	9606.ENSI	0	0	0	0
SHC1	SYK	1860266	1856371	9606.ENSI	9606.ENSI	0	0	0	0
SHC1	PDGFRB	1860266	1844996	9606.ENSI	9606.ENSI	0	0	0	0
SHC1	KIT	1860266	1847112	9606.ENSI	9606.ENSI	0	0	0	0
SHC1	MAPK1	1860266	1842512	9606.ENSI	9606.ENSI	0	0	0	0
SHC1	FGFR1	1860266	1859710	9606.ENSI	9606.ENSI	0	0	0	0
SHC1	MAPK14	1860266	1843222	9606.ENSI	9606.ENSI	0	0	0	0
SHC1	AKT1	1860266	1846136	9606.ENSI	9606.ENSI	0	0	0	0
SHC1	PTPN11	1860266	1852389	9606.ENSI	9606.ENSI	0	0	0	0.614
SHC1	ITGB3	1860266	1845040	9606.ENSI	9606.ENSI	0	0	0	0
SHC1	HRAS	1860266	1849235	9606.ENSI	9606.ENSI	0	0	0	0
SHC1	PIK3CA	1860266	1845446	9606.ENSI	9606.ENSI	0	0	0	0
SHC1	IL6	1860266	1844631	9606.ENSI	9606.ENSI	0	0	0	0
SHC1	APP	1860266	1846936	9606.ENSI	9606.ENSI	0	0	0	0
SHC1	TGFBR1	1860266	1856216	9606.ENSI	9606.ENSI	0	0	0	0
SHC1	ITGB1	1860266	1848520	9606.ENSI	9606.ENSI	0	0	0	0
SHC1	S1PR1	1860266	1848727	9606.ENSI	9606.ENSI	0	0	0	0
SHC1	ESR1	1860266	1842435	9606.ENSI	9606.ENSI	0	0	0	0
SHC1	TP53	1860266	1846083	9606.ENSI	9606.ENSI	0	0	0	0

SHC1	HLA-DRB1	1860266	1853944	9606.ENSI	9606.ENSI	0	0	0	0
SHH	EGFR	1847842	1846445	9606.ENSI	9606.ENSI	0	0	0	0
SHH	CCND1	1847842	1843117	9606.ENSI	9606.ENSI	0	0	0	0
SHH	AKT1	1847842	1846136	9606.ENSI	9606.ENSI	0	0	0	0
SHH	TP53	1847842	1846083	9606.ENSI	9606.ENSI	0	0	0	0
STAT3	CCND1	1845584	1843117	9606.ENSI	9606.ENSI	0	0	0	0
STAT3	PDGFRB	1845584	1844996	9606.ENSI	9606.ENSI	0	0	0	0
STAT3	IL6	1845584	1844631	9606.ENSI	9606.ENSI	0	0	0	0
STAT3	MAPK1	1845584	1842512	9606.ENSI	9606.ENSI	0	0	0	0
STAT3	MAPK14	1845584	1843222	9606.ENSI	9606.ENSI	0	0	0	0
STAT3	RAF1	1845584	1844086	9606.ENSI	9606.ENSI	0	0	0	0
STAT3	PIK3CA	1845584	1845446	9606.ENSI	9606.ENSI	0	0	0	0
STAT3	MMP2	1845584	1842719	9606.ENSI	9606.ENSI	0	0	0	0
STAT3	IL1B	1845584	1845335	9606.ENSI	9606.ENSI	0	0	0	0
SYK	STAT3	1856371	1845584	9606.ENSI	9606.ENSI	0	0	0	0
SYK	PIK3CG	1856371	1853801	9606.ENSI	9606.ENSI	0	0	0	0
SYK	ITGB3	1856371	1845040	9606.ENSI	9606.ENSI	0	0	0	0
SYK	PTPN11	1856371	1852389	9606.ENSI	9606.ENSI	0	0	0	0.615
SYK	MAPK14	1856371	1843222	9606.ENSI	9606.ENSI	0	0	0	0.631
SYK	PIK3CA	1856371	1845446	9606.ENSI	9606.ENSI	0	0	0	0
SYK	MAPT	1856371	1852372	9606.ENSI	9606.ENSI	0	0	0	0
TBXA2R	ADRA1B	1859713	1848870	9606.ENSI	9606.ENSI	0	0	0	0
TBXA2R	ADRA1D	1859713	1857034	9606.ENSI	9606.ENSI	0	0	0	0
TBXA2R	F2R	1859713	1850333	9606.ENSI	9606.ENSI	0	0	0	0
TBXA2R	PTGIR	1859713	1847278	9606.ENSI	9606.ENSI	0	0	0	0.724
TBXA2R	EDNRB	1859713	1856642	9606.ENSI	9606.ENSI	0	0	0	0
TBXA2R	HTR2A	1859713	1856916	9606.ENSI	9606.ENSI	0	0	0	0
TBXA2R	AGTR1	1859713	1846323	9606.ENSI	9606.ENSI	0	0	0	0
TBXA2R	SYK	1859713	1856371	9606.ENSI	9606.ENSI	0	0	0	0
TBXA2R	EDNRA	1859713	1849748	9606.ENSI	9606.ENSI	0	0	0	0
TBXA2R	PTGER1	1859713	1847360	9606.ENSI	9606.ENSI	0	0	0	0.66
TBXA2R	BDKRB2	1859713	1848988	9606.ENSI	9606.ENSI	0	0	0	0
TBXA2R	CHRM1	1859713	1848849	9606.ENSI	9606.ENSI	0	0	0	0
TBXA2R	PRKCH	1859713	1851111	9606.ENSI	9606.ENSI	0	0	0	0
TBXA2R	HTR2B	1859713	1844597	9606.ENSI	9606.ENSI	0	0	0	0
TBXA2R	APP	1859713	1846936	9606.ENSI	9606.ENSI	0	0	0	0
TBXA2R	PIK3CA	1859713	1845446	9606.ENSI	9606.ENSI	0	0	0	0
TBXA2R	FFAR1	1859713	1843825	9606.ENSI	9606.ENSI	0	0	0	0
TBXA2R	ADRA1A	1859713	1857247	9606.ENSI	9606.ENSI	0	0	0	0
TERT	AKT1	1849199	1846136	9606.ENSI	9606.ENSI	0	0	0	0
TERT	TP53	1849199	1846083	9606.ENSI	9606.ENSI	0	0	0	0
TERT	RPS6KB1	1849199	1843035	9606.ENSI	9606.ENSI	0	0	0	0
TERT	CCND1	1849199	1843117	9606.ENSI	9606.ENSI	0	0	0	0
TERT	ESR1	1849199	1842435	9606.ENSI	9606.ENSI	0	0	0	0
TERT	EGFR	1849199	1846445	9606.ENSI	9606.ENSI	0	0	0	0
TERT	MAPK1	1849199	1842512	9606.ENSI	9606.ENSI	0	0	0	0
TGFBR1	SERPINE1	1856216	1842959	9606.ENSI	9606.ENSI	0	0	0	0
TGFBR1	RPS6KB1	1856216	1843035	9606.ENSI	9606.ENSI	0	0	0	0.617
THRA	ESR1	1845577	1842435	9606.ENSI	9606.ENSI	0	0	0	0.653
THRA	PPARA	1845577	1845205	9606.ENSI	9606.ENSI	0	0	0	0.685
THRA	NR1H2	1845577	1844246	9606.ENSI	9606.ENSI	0	0	0	0.748
TNF	MMP9	1860103	1855661	9606.ENSI	9606.ENSI	0	0	0	0
TNF	PTGS2	1860103	1854602	9606.ENSI	9606.ENSI	0	0	0	0
TNF	IL1B	1860103	1845335	9606.ENSI	9606.ENSI	0	0	0	0

TNF	BCL2	1860103	1851168	9606.ENSI	9606.ENSI	0	0	0	0
TNF	AKT1	1860103	1846136	9606.ENSI	9606.ENSI	0	0	0	0
TNF	TP53	1860103	1846083	9606.ENSI	9606.ENSI	0	0	0	0
TNF	IRS1	1860103	1848682	9606.ENSI	9606.ENSI	0	0	0	0
TNNT2	TNNI3	1854566	1852512	9606.ENSI	9606.ENSI	0	0	0	0
TP53	MAPK14	1846083	1843222	9606.ENSI	9606.ENSI	0	0	0	0
TP53	MAPK1	1846083	1842512	9606.ENSI	9606.ENSI	0	0	0	0
TP53	CSNK2A1	1846083	1842650	9606.ENSI	9606.ENSI	0	0	0	0
TP53	MMP2	1846083	1842719	9606.ENSI	9606.ENSI	0	0	0	0
TP53	ABCB1	1846083	1845794	9606.ENSI	9606.ENSI	0	0	0	0
TP53	SERPINE1	1846083	1842959	9606.ENSI	9606.ENSI	0	0	0	0
TP53	CDK4	1846083	1844551	9606.ENSI	9606.ENSI	0	0	0	0
TP53	CCND1	1846083	1843117	9606.ENSI	9606.ENSI	0	0	0	0
TP53	VHL	1846083	1844441	9606.ENSI	9606.ENSI	0	0	0	0
TP53	ESR1	1846083	1842435	9606.ENSI	9606.ENSI	0	0	0	0
TP53	STAT3	1846083	1845584	9606.ENSI	9606.ENSI	0	0	0	0
TP53	CTSD	1846083	1843439	9606.ENSI	9606.ENSI	0	0	0	0
TP53	NR3C1	1846083	1843294	9606.ENSI	9606.ENSI	0	0	0	0
TP53	PIK3CA	1846083	1845446	9606.ENSI	9606.ENSI	0	0	0	0
TP53	IL6	1846083	1844631	9606.ENSI	9606.ENSI	0	0	0	0
TRPV1	EGFR	1858755	1846445	9606.ENSI	9606.ENSI	0	0	0	0
TRPV1	MMP1	1858755	1850481	9606.ENSI	9606.ENSI	0	0	0	0
TSHR	STAT3	1847934	1845584	9606.ENSI	9606.ENSI	0	0	0	0
TSHR	PTGIR	1847934	1847278	9606.ENSI	9606.ENSI	0	0	0	0
VDR	TP53	1862386	1846083	9606.ENSI	9606.ENSI	0	0	0	0
VDR	ESR1	1862386	1842435	9606.ENSI	9606.ENSI	0	0	0	0.649
VDR	AR	1862386	1856164	9606.ENSI	9606.ENSI	0	0	0	0.632
VDR	PPARA	1862386	1845205	9606.ENSI	9606.ENSI	0	0	0	0.701
VDR	ESR2	1862386	1852756	9606.ENSI	9606.ENSI	0	0	0	0.644
VDR	PPARG	1862386	1847090	9606.ENSI	9606.ENSI	0	0	0	0.715
VDR	THRA	1862386	1845577	9606.ENSI	9606.ENSI	0	0	0	0.695
VDR	NR1H2	1862386	1844246	9606.ENSI	9606.ENSI	0	0	0	0.695
VDR	PPARD	1862386	1849364	9606.ENSI	9606.ENSI	0	0	0	0.706

coexpres	experime	database_	automate	predicti	combined_score
0	0	0.9	0.909	0	0.99
0	0	0.9	0.946	0	0.994
0	0	0.9	0.96	0	0.926
0	0	0.9	0.07	0	0.903
0	0.57	0.9	0.904	0	0.957
0	0.57	0.9	0.332	0	0.955
0	0	0.9	0.351	0	0.911
0	0	0.9	0.25	0	0.907
0	0	0.9	0.285	0	0.906
0	0	0.9	0.191	0	0.904
0	0	0.9	0.123	0	0.903
0	0	0.9	0.065	0	0.902
0	0	0.9	0.097	0	0.902
0	0	0.9	0	0	0.9
0	0	0.9	0	0	0.9
0	0	0.9	0	0	0.9
0	0	0.9	0	0	0.9
0	0	0.9	0	0	0.9
0	0	0.9	0.729	0	0.913
0	0	0.9	0.163	0	0.912
0	0	0.9	0.234	0	0.907
0	0	0.9	0.262	0	0.906
0	0	0.9	0.178	0	0.905
0	0	0.9	0.177	0	0.904
0	0	0.9	0.065	0	0.902
0	0	0.9	0.053	0	0.9
0	0	0.9	0	0	0.9
0	0	0.9	0	0	0.9
0	0	0.9	0.269	0	0.907
0	0	0.9	0.274	0	0.907
0	0	0.9	0.228	0	0.906
0	0	0.9	0.243	0	0.905
0	0	0.9	0.094	0	0.905
0	0	0.9	0.748	0	0.904
0	0	0.9	0.074	0	0.903
0	0	0.9	0.121	0	0.903
0	0	0.9	0.065	0	0.902
0	0	0.9	0	0	0.9
0	0	0.9	0	0	0.9
0	0	0.9	0	0	0.9
0	0	0.9	0	0	0.9
0	0	0.9	0.398	0	0.91
0	0	0.9	0.308	0	0.905
0	0	0.9	0.199	0	0.904
0	0	0.9	0	0	0.9
0	0	0.9	0.047	0	0.9
0	0	0.9	0	0	0.9
0	0	0.9	0	0	0.9
0	0.149	0.9	0.876	0	0.915
0	0	0.9	0.064	0	0.902
0	0	0.9	0	0	0.9
0	0.576	0.9	0.075	0	0.957
0	0.16	0.9	0.306	0	0.936

0	0.564	0	0.806	0	0.911
0	0	0.9	0.213	0	0.906
0	0	0.9	0.252	0	0.906
0	0	0.9	0.045	0	0.9
0	0.576	0.9	0.14	0	0.957
0	0	0.9	0	0	0.9
0	0	0.9	0	0	0.9
0	0.668	0.9	0.991	0	0.999
0	0.576	0.9	0.96	0	0.998
0	0.984	0.9	0.989	0	0.998
0.117	0.969	0.9	0.993	0	0.997
0	0	0	0.976	0	0.977
0	0	0	0.974	0	0.975
0	0	0	0.973	0	0.974
0	0.576	0.9	0.38	0	0.971
0	0.945	0	0.976	0	0.963
0	0	0	0.949	0	0.949
0	0	0	0.946	0	0.947
0	0.101	0.9	0.95	0	0.94
0	0	0	0.923	0	0.923
0	0	0.9	0.937	0	0.993
0	0	0	0.96	0	0.96
0	0.053	0.9	0.581	0	0.956
0	0	0	0.926	0	0.926
0	0	0	0.923	0	0.923
0	0	0	0.901	0	0.902
0	0.591	0	0.973	0	0.988
0	0	0.9	0.719	0	0.97
0	0	0.9	0.276	0	0.924
0	0	0.9	0.206	0	0.917
0	0	0.9	0.197	0	0.916
0	0	0.9	0.176	0	0.914
0	0	0.9	0.173	0	0.913
0	0	0.9	0	0	0.9
0	0	0.9	0	0	0.9
0	0.812	0.9	0.937	0	0.998
0	0.926	0	0.938	0	0.995
0	0.914	0	0.942	0	0.994
0	0.917	0	0.913	0	0.992
0	0.879	0.9	0.405	0	0.992
0	0.812	0	0.943	0	0.988
0	0.576	0.9	0.908	0	0.968
0	0.576	0.9	0.198	0	0.963
0	0.576	0.9	0.889	0	0.962
0	0.097	0.9	0.479	0	0.948
0	0	0.9	0.342	0	0.931
0	0.099	0.9	0.266	0	0.928
0	0	0	0.923	0	0.923
0	0	0.9	0.729	0	0.921
0	0	0.9	0.565	0	0.92
0	0	0.9	0.581	0	0.92
0	0	0.9	0.416	0	0.914
0	0	0.9	0.243	0	0.907
0	0	0.9	0.191	0	0.905

0	0.983	0	0.953	0	0.999
0	0.983	0.9	0.989	0	0.999
0	0	0.9	0.981	0	0.998
0	0.807	0.9	0.898	0	0.997
0	0.564	0	0.945	0	0.975
0	0	0	0.974	0	0.974
0	0.576	0	0.941	0	0.974
0	0.576	0	0.919	0	0.964
0	0.576	0	0.896	0	0.954
0	0	0	0.943	0	0.943
0	0	0	0.942	0	0.942
0	0	0	0.928	0	0.929
0	0.576	0.9	0.503	0	0.959
0	0	0.9	0.243	0	0.921
0	0	0.9	0.126	0	0.908
0	0	0.9	0.105	0	0.906
0	0	0.9	0.126	0	0.902
0	0	0.9	0	0	0.9
0	0	0.9	0	0	0.9
0	0	0.9	0	0	0.9
0	0	0.9	0	0	0.9
0	0	0.9	0	0	0.9
0	0	0.9	0	0	0.9
0	0.999	0.9	0.636	0	0.999
0.071	0.871	0.9	0.767	0	0.99
0	0.938	0	0.964	0	0.959
0	0.101	0.9	0	0	0.906
0	0.929	0	0.938	0	0.995
0	0.569	0.9	0.543	0	0.978
0	0.576	0.9	0.217	0	0.963
0.088	0.569	0	0.911	0	0.962
0	0	0.9	0.151	0	0.911
0	0	0.9	0.674	0	0.966
0	0	0.9	0.323	0	0.929
0	0	0.9	0.28	0	0.925
0	0	0.9	0.257	0	0.922
0	0	0.9	0.203	0	0.916
0	0	0.9	0.202	0	0.916
0	0	0.9	0.19	0	0.915
0	0	0.9	0.19	0	0.915
0	0	0.9	0.185	0	0.915
0	0	0.9	0.18	0	0.914
0	0	0.9	0.167	0	0.913
0	0	0.9	0.159	0	0.912
0	0	0.9	0.139	0	0.91
0	0	0.9	0.123	0	0.908
0	0	0.9	0.101	0	0.906
0	0	0.9	0.1	0	0.906
0	0	0.9	0.089	0	0.905
0	0	0.9	0.073	0	0.903
0	0	0.9	0.063	0	0.902
0	0	0.9	0.063	0	0.902
0	0	0.9	0	0	0.9
0	0	0.9	0	0	0.9

0	0.917	0.9	0.96	0	0.999
0	0.573	0	0.882	0	0.947
0	0.803	0	0.846	0	0.968
0	0	0.9	0.163	0	0.912
0	0	0.9	0	0	0.9
0	0.999	0.9	0.995	0	0.999
0	0	0.9	0.086	0	0.904
0	0.999	0.9	0.993	0	0.999
0	0.842	0.9	0.987	0	0.984
0	0	0.9	0.155	0	0.911
0	0	0.9	0.372	0	0.91
0	0	0.9	0.136	0	0.909
0	0	0.9	0.102	0	0.906
0	0	0.9	0.191	0	0.905
0	0	0.9	0.196	0	0.905
0	0	0.9	0.169	0	0.904
0	0	0.9	0.073	0	0.903
0	0	0.9	0.073	0	0.903
0	0	0.9	0	0	0.9
0	0	0.9	0.109	0	0.907
0	0	0.9	0.188	0	0.905
0	0	0.9	0.073	0	0.903
0	0	0.9	0.071	0	0.903
0.071	0	0.9	0.7	0	0.969
0	0	0.9	0.321	0	0.929
0	0	0.9	0.652	0	0.924
0	0	0	0.956	0	0.957
0	0	0.9	0.898	0	0.945
0	0	0.9	0.867	0	0.932
0	0	0	0.91	0	0.91
0	0.361	0.9	0.43	0	0.96
0	0	0	0.911	0	0.911
0	0	0.9	0.405	0	0.938
0	0	0	0.926	0	0.926
0	0	0.9	0.12	0	0.908
0	0.576	0.8	0.829	0	0.984
0	0	0.9	0.434	0	0.911
0	0	0.9	0.091	0	0.905
0	0	0.9	0.084	0	0.904
0	0	0.9	0.159	0	0.904
0	0	0.9	0	0	0.9
0	0	0.9	0.324	0	0.908
0	0	0.9	0.181	0	0.905
0	0	0.9	0.888	0	0.904
0	0	0.9	0.074	0	0.903
0	0	0.9	0.055	0	0.901
0	0	0.9	0.046	0	0.9
0	0	0.9	0.359	0	0.933
0	0	0.9	0.33	0	0.93
0	0	0.9	0.198	0	0.916
0	0	0.9	0.452	0	0.914
0	0	0.9	0.113	0	0.907
0	0	0.9	0.18	0	0.905
0	0	0.9	0.172	0	0.905

0	0	0.9	0.073	0	0.903
0	0	0.9	0.053	0	0.9
0	0	0.9	0	0	0.9
0	0	0.9	0	0	0.9
0	0	0.9	0.223	0	0.918
0	0	0.9	0.418	0	0.913
0	0	0.9	0.35	0	0.912
0	0	0.9	0.163	0	0.912
0	0	0.9	0.326	0	0.911
0	0	0.9	0.312	0	0.91
0	0	0.9	0.092	0	0.905
0	0	0.9	0.947	0	0.904
0	0	0.9	0.146	0	0.904
0	0	0.9	0.073	0	0.903
0	0	0.9	0	0	0.9
0	0	0.9	0	0	0.9
0	0.999	0.9	0.985	0	0.999
0	0	0.9	0.974	0	0.997
0	0.924	0	0.973	0	0.997
0	0	0.9	0.891	0	0.988
0	0.693	0.9	0.855	0	0.978
0	0	0	0.963	0	0.963
0	0	0.9	0.625	0	0.96
0	0	0	0.928	0	0.928
0	0.884	0	0.971	0	0.926
0	0.149	0	0.888	0	0.901
0	0.889	0.9	0.99	0	0.999
0	0.999	0.9	0.985	0	0.999
0	0.934	0	0.974	0	0.998
0	0.604	0	0.993	0	0.997
0	0.807	0.8	0.689	0	0.987
0	0.812	0	0.731	0	0.947
0	0	0	0.93	0	0.93
0	0.104	0	0.896	0	0.903
0	0	0.9	0.295	0	0.926
0	0.088	0.9	0.172	0	0.917
0	0.988	0.9	0.981	0	0.998
0	0.567	0.9	0.472	0	0.962
0	0.601	0.8	0.259	0	0.935
0	0	0.9	0.593	0	0.919
0	0	0.9	0.417	0	0.914
0	0	0.9	0.243	0	0.907
0	0	0.9	0.251	0	0.906
0	0.812	0	0.88	0	0.977
0.115	0.576	0.9	0.299	0	0.962
0	0	0.9	0.517	0	0.949
0	0	0.9	0.509	0	0.948
0	0	0.9	0.407	0	0.938
0	0	0	0.929	0	0.929
0	0	0	0.924	0	0.924
0	0	0.9	0.235	0	0.92
0	0	0.9	0.163	0	0.912
0	0	0	0.912	0	0.912
0	0	0.9	0.142	0	0.91

0	0	0.9	0.145	0	0.91
0	0	0.9	0.077	0	0.903
0	0	0.9	0	0	0.9
0	0.98	0.9	0.886	0	0.999
0	0.572	0.8	0.538	0	0.956
0	0	0.9	0.288	0	0.925
0	0	0.9	0.458	0	0.914
0	0	0.9	0.168	0	0.913
0	0	0.9	0.16	0	0.912
0	0	0.9	0.129	0	0.909
0	0	0.9	0.341	0	0.908
0	0	0.9	0.092	0	0.905
0	0	0.9	0.113	0	0.902
0	0	0.9	0.066	0	0.902
0	0	0.9	0	0	0.9
0	0	0.9	0.045	0	0.9
0	0.326	0.8	0.564	0	0.936
0.099	0	0	0.9	0	0.906
0	0.088	0.8	0.53	0	0.906
0	0.82	0.9	0.975	0	0.999
0	0.823	0.9	0.989	0	0.983
0	0.088	0.9	0.472	0	0.947
0	0.09	0.9	0.917	0	0.913
0	0.076	0.9	0.744	0	0.914
0	0.076	0.9	0.605	0	0.911
0	0	0.9	0.764	0	0.901
0	0	0.9	0.649	0	0.901
0	0.076	0.9	0.718	0	0.915
0	0.076	0.9	0.679	0	0.913
0	0	0.9	0.59	0	0.901
0	0	0.9	0.647	0	0.901
0	0	0.9	0.63	0	0.901
0	0.076	0.9	0.909	0	0.917
0	0.076	0.9	0.885	0	0.916
0	0	0.9	0.733	0	0.901
0.066	0.109	0.9	0.84	0	0.92
0	0.076	0.9	0.771	0	0.914
0	0	0.9	0.169	0	0.9
0	0.109	0.9	0.843	0	0.914
0	0	0.9	0.823	0	0.914
0	0.082	0.9	0.794	0	0.911
0	0.082	0.9	0.609	0	0.91
0	0.082	0.9	0.683	0	0.91
0	0	0.9	0.73	0	0.91
0	0	0.9	0.074	0	0.903
0	0	0.9	0	0	0.9
0	0	0.9	0.583	0	0.956
0	0.999	0.9	0.985	0	0.999
0	0.964	0.9	0.981	0	0.999
0	0.984	0.9	0.881	0	0.999
0	0.59	0.9	0.988	0	0.999
0	0.567	0	0.982	0	0.992
0	0.607	0.9	0.52	0	0.979
0	0.113	0.9	0.665	0	0.967

0	0.113	0.9	0.665	0	0.967
0.071	0.346	0.8	0.737	0	0.963
0	0.074	0	0.944	0	0.947
0	0	0.9	0.463	0	0.944
0	0	0.9	0.458	0	0.943
0	0.157	0.9	0.297	0	0.935
0	0	0.9	0.183	0	0.914
0	0	0.9	0.33	0	0.911
0	0	0.9	0.33	0	0.911
0	0	0.9	0.96	0	0.907
0	0	0.9	0.192	0	0.905
0	0	0.9	0.181	0	0.905
0	0	0.9	0.203	0	0.905
0	0	0.9	0.07	0	0.903
0	0	0.9	0.144	0	0.902
0	0	0.9	0.055	0	0.901
0	0	0.9	0.041	0	0.9
0	0	0.9	0	0	0.9
0	0	0.9	0.065	0	0.902
0	0	0.9	0.897	0	0.989
0.158	0	0	0.973	0	0.976
0.066	0.576	0	0.91	0	0.961
0	0	0	0.943	0	0.943
0	0	0	0.937	0	0.937
0	0	0	0.927	0	0.927
0	0	0	0.923	0	0.923
0	0	0	0.912	0	0.912
0	0.088	0.9	0.764	0	0.976
0	0.227	0.9	0.958	0	0.948
0	0.251	0.9	0.771	0	0.944
0	0.998	0.9	0.996	0	0.999
0	0.567	0.9	0.979	0	0.999
0	0.993	0.9	0.876	0	0.999
0	0.576	0	0.992	0	0.996
0	0.576	0.9	0.388	0	0.971
0	0.567	0	0.902	0	0.955
0	0.237	0.8	0.669	0	0.945
0	0	0	0.927	0	0.927
0	0	0	0.923	0	0.923
0	0	0.9	0.163	0	0.912
0	0.104	0.8	0.516	0	0.905
0	0.576	0.9	0.242	0	0.965
0	0	0.9	0.525	0	0.95
0	0	0.9	0.378	0	0.935
0	0	0.9	0.283	0	0.925
0	0	0.9	0.125	0	0.908
0	0	0.9	0.053	0	0.901
0	0.576	0.9	0.289	0	0.967
0	0	0.9	0.472	0	0.944
0	0.101	0.9	0.267	0	0.928
0	0.968	0.9	0.976	0	0.999
0	0.985	0.9	0.992	0	0.999
0	0.92	0.9	0.974	0	0.999
0	0	0.9	0.943	0	0.994

0	0.819	0.9	0.932	0	0.987
0	0.812	0.9	0.207	0	0.983
0	0.593	0.9	0.744	0	0.969
0	0.088	0.9	0.643	0	0.964
0	0.812	0	0.793	0	0.959
0	0.113	0.9	0.572	0	0.958
0	0	0.9	0.359	0	0.933
0	0	0	0.929	0	0.93
0	0.194	0.9	0.457	0	0.928
0	0	0.9	0.228	0	0.919
0	0.576	0	0.811	0	0.916
0	0.088	0.8	0.575	0	0.915
0	0	0.9	0.096	0	0.905
0	0.851	0	0.962	0	0.904
0	0	0.9	0.068	0	0.902
0	0.1	0.9	0.943	0	0.994
0	0.963	0.9	0.981	0	0.996
0	0.576	0.9	0.271	0	0.966
0	0	0	0.909	0	0.909
0	0.111	0.9	0.612	0	0.962
0	0.149	0.9	0.464	0	0.95
0	0.101	0.9	0.446	0	0.921
0	0	0.9	0.725	0	0.971
0	0	0.9	0.508	0	0.948
0	0	0.9	0.748	0	0.905
0	0	0.9	0.962	0	0.996
0	0	0.9	0.811	0	0.98
0	0	0.9	0.659	0	0.964
0	0	0.9	0.215	0	0.918
0	0	0.9	0.813	0	0.905
0	0	0.9	0.493	0	0.902
0	0.919	0.9	0.344	0	0.994
0	0.982	0.8	0.551	0	0.996
0	0.601	0.9	0.395	0	0.973
0	0	0.9	0.566	0	0.903
0	0	0	0.901	0	0.901
0	0.912	0	0.885	0	0.989
0	0.576	0	0.901	0	0.956
0	0.812	0	0.703	0	0.941
0	0.576	0	0.823	0	0.921
0	0.992	0.9	0.986	0	0.999
0	0.999	0.9	0.789	0	0.999
0	0.999	0.9	0.99	0	0.999
0	0.999	0.9	0.987	0	0.999
0	0.638	0.9	0.842	0	0.993
0	0.194	0.9	0.758	0	0.978
0	0	0.9	0.478	0	0.945
0	0	0.9	0.682	0	0.966
0	0	0.9	0.874	0	0.986
0	0	0	0.928	0	0.928
0	0	0	0.928	0	0.928
0.113	0	0.9	0.914	0	0.924
0	0	0.9	0.216	0	0.918
0	0	0	0.915	0	0.915

0	0	0	0.912	0	0.912
0	0	0	0.928	0	0.928
0	0.576	0.9	0.962	0	0.998
0	0	0	0.952	0	0.952
0	0	0	0.949	0	0.949
0	0	0	0.947	0	0.947
0	0	0	0.942	0	0.942
0	0	0	0.94	0	0.94
0.066	0	0	0.937	0	0.939
0	0	0.9	0.358	0	0.933
0.113	0	0.9	0.982	0	0.93
0	0	0	0.928	0	0.929
0	0	0	0.926	0	0.926
0	0	0.9	0.989	0	0.905
0	0	0	0.902	0	0.902
0	0.82	0.9	0.964	0	0.999
0	0.999	0.9	0.995	0	0.999
0	0.999	0.9	0.996	0	0.999
0	0.917	0.9	0.942	0	0.999
0	0.999	0.9	0.997	0	0.999
0	0.999	0.9	0.995	0	0.999
0.168	0.999	0.9	0.996	0	0.999
0	0.706	0.9	0.881	0	0.996
0	0.576	0	0.972	0	0.988
0	0.293	0.9	0.823	0	0.986
0	0.292	0.9	0.731	0	0.979
0	0	0.9	0.772	0	0.976
0	0.292	0.9	0.898	0	0.951
0	0	0	0.949	0	0.949
0	0.088	0	0.943	0	0.947
0.073	0	0	0.929	0	0.932
0	0	0	0.912	0	0.912
0	0	0.8	0.566	0	0.909
0	0.088	0	0.901	0	0.906
0	0.318	0.8	0.336	0	0.901
0	0	0.9	0.118	0	0.908
0	0	0.9	0.247	0	0.907
0	0	0.8	0.648	0	0.926
0	0	0.9	0.206	0	0.917
0	0	0.9	0.701	0	0.968
0	0	0.9	0.331	0	0.93
0	0.812	0.9	0.263	0	0.981
0	0.576	0.9	0.954	0	0.966
0	0	0.9	0.503	0	0.948
0	0	0.9	0.959	0	0.902
0	0	0	0.956	0	0.957
0	0	0.9	0.277	0	0.924
0	0	0.9	0.487	0	0.914
0	0.92	0.9	0.973	0	0.999
0	0.996	0.9	0.928	0	0.999
0	0	0.9	0.859	0	0.985
0	0.576	0.9	0.326	0	0.968
0	0	0.9	0.243	0	0.921
0	0	0.9	0.18	0	0.905

0	0.898	0	0.603	0	0.921
0	0.663	0.9	0.88	0	0.995
0	0.81	0.9	0.539	0	0.99
0	0.596	0.9	0.556	0	0.98
0	0	0.9	0.652	0	0.963
0	0.088	0.9	0.53	0	0.953
0	0.088	0.9	0.3	0	0.93
0	0	0.8	0.662	0	0.929
0	0	0.9	0.291	0	0.926
0	0	0.9	0.163	0	0.912
0	0	0.9	0.069	0	0.902
0	0	0.9	0	0	0.9
0	0	0.9	0	0	0.9
0	0.96	0.8	0.58	0	0.996
0	0.251	0.9	0.915	0	0.993
0	0.954	0.8	0.347	0	0.993
0	0.576	0.8	0.802	0	0.981
0	0.245	0.8	0.829	0	0.972
0	0	0.8	0.656	0	0.928
0	0	0.9	0.908	0	0.915
0	0	0.9	0.879	0	0.987
0	0	0.9	0.584	0	0.956
0	0	0.9	0.394	0	0.936
0	0	0.9	0	0	0.9
0	0.576	0.9	0.98	0	0.999
0.141	0	0.9	0.757	0	0.977
0	0	0.9	0.266	0	0.923
0	0.601	0.9	0.317	0	0.97
0	0	0.9	0.71	0	0.969
0	0	0	0.942	0	0.942
0	0	0	0.937	0	0.937
0	0	0.9	0.892	0	0.93
0	0	0.9	0.7	0	0.924
0	0	0.9	0.509	0	0.913
0	0	0.9	0.394	0	0.912
0	0	0.9	0.267	0	0.906
0	0	0.9	0.822	0	0.902
0	0	0.9	0.826	0	0.902
0	0.915	0.9	0.639	0	0.993
0	0	0.9	0.721	0	0.97
0	0	0.9	0.383	0	0.935
0	0	0.9	0.35	0	0.932
0	0	0.9	0.882	0	0.924
0	0	0	0.923	0	0.923
0	0	0	0.912	0	0.912
0	0	0.9	0.293	0	0.908
0	0	0	0.903	0	0.903
0	0	0.9	0.915	0	0.903
0	0.923	0.9	0.354	0	0.992
0	0.884	0.9	0.885	0	0.991
0	0.812	0.9	0.091	0	0.981
0	0.677	0.9	0.464	0	0.981
0	0.617	0.9	0.512	0	0.979
0	0.576	0.9	0.916	0	0.97

0	0.812	0.8	0.124	0	0.964
0	0.602	0.9	0.264	0	0.962
0	0.576	0.9	0.101	0	0.958
0	0.149	0.9	0.453	0	0.949
0	0.347	0.9	0.69	0	0.948
0	0.576	0	0.878	0	0.946
0	0.251	0.9	0.342	0	0.946
0	0.157	0.9	0.397	0	0.944
0	0.215	0.9	0.584	0	0.925
0	0.101	0.9	0.289	0	0.915
0	0.101	0.9	0.244	0	0.913
0	0	0.9	0.077	0	0.903
0	0	0.9	0.765	0	0.901
0	0.576	0.9	0.252	0	0.959
0	0.215	0.9	0.439	0	0.924
0	0	0.9	0.462	0	0.916
0	0	0.9	0.36	0	0.912
0	0.347	0.9	0.559	0	0.945
0	0.157	0.9	0.146	0	0.921
0	0.965	0.9	0.994	0	0.999
0	0.824	0	0.939	0	0.988
0	0	0.9	0.734	0	0.972
0	0	0.9	0.654	0	0.963
0	0	0.9	0.291	0	0.926
0	0	0.9	0.211	0	0.917
0	0	0.9	0.071	0	0.903
0	0	0.9	0	0	0.9
0	0	0.9	0.043	0	0.9
0	0	0.9	0	0	0.9
0	0	0.9	0	0	0.9
0	0	0.9	0	0	0.9
0	0	0.9	0	0	0.93
0	0.961	0	0.94	0	0.997
0	0	0.9	0.871	0	0.986
0	0	0.9	0.844	0	0.983
0	0	0	0.942	0	0.942
0	0	0	0.939	0	0.94
0	0	0	0.916	0	0.916
0.073	0	0	0.912	0	0.915
0	0	0	0.908	0	0.908
0	0.149	0	0.891	0	0.904
0	0.919	0.9	0.94	0	0.999
0	0.957	0.9	0.982	0	0.999
0	0.917	0.9	0.946	0	0.999
0	0.991	0.9	0.977	0	0.999
0	0.999	0.9	0.952	0	0.999
0	0.583	0.9	0.926	0	0.996
0	0.841	0.9	0.551	0	0.992
0	0	0.9	0.921	0	0.991
0	0	0.9	0.882	0	0.987
0	0.576	0	0.967	0	0.985
0.069	0.361	0.9	0.732	0	0.981
0	0.253	0.9	0.775	0	0.981
0	0	0.9	0.815	0	0.98

0	0	0	0.929	0	0.93
0	0.588	0	0.787	0	0.909
0.071	0.987	0.9	0.862	0	0.999
0	0	0	0.922	0	0.922
0	0	0	0.912	0	0.912
0	0	0	0.909	0	0.909
0	0.986	0	0.445	0	0.992
0	0.326	0.9	0.705	0	0.978
0	0.594	0.9	0.378	0	0.972
0	0.088	0.9	0.572	0	0.957
0	0.104	0.9	0.289	0	0.93
0	0.881	0	0.75	0	0.914
0	0.974	0	0.987	0	0.999
0	0.997	0.9	0.986	0	0.999
0	0.999	0	0.882	0	0.999
0	0.81	0	0.99	0	0.998
0	0.671	0.9	0.702	0	0.973
0	0.999	0.9	0.992	0	0.999
0	0.802	0.9	0.933	0	0.998
0	0.672	0.9	0.917	0	0.997
0	0.808	0	0.545	0	0.909
0	0.576	0.9	0.913	0	0.996
0	0	0.9	0.859	0	0.985
0	0.576	0.9	0.347	0	0.969
0	0	0.9	0.194	0	0.915
0	0	0.9	0.041	0	0.9
0	0	0.9	0	0	0.9
0	0	0.9	0.046	0	0.9
0	0.815	0.9	0.964	0	0.999
0	0.967	0.9	0.933	0	0.999
0	0.921	0.9	0.9	0	0.999
0	0.983	0.9	0.985	0	0.999
0	0.969	0.9	0.907	0	0.999
0	0.999	0.9	0.989	0	0.999
0	0.576	0.9	0.967	0	0.998
0	0.641	0.9	0.906	0	0.996
0	0.594	0.9	0.925	0	0.996
0	0.648	0.8	0.924	0	0.994
0	0.237	0.9	0.901	0	0.991
0	0.808	0.9	0.58	0	0.991
0	0.603	0.8	0.893	0	0.99
0	0.57	0.9	0.806	0	0.99
0	0	0.9	0.876	0	0.987
0	0.564	0.9	0.959	0	0.971
0	0.576	0.9	0.217	0	0.963
0	0	0.9	0.638	0	0.962
0	0	0.9	0.628	0	0.961
0	0	0.9	0.458	0	0.943
0	0.917	0	0.309	0	0.94
0	0.105	0.9	0.214	0	0.923
0	0	0.9	0.234	0	0.92
0	0	0.9	0.081	0	0.904
0	0.808	0	0.509	0	0.901
0	0	0	0.9	0	0.9

0	0	0.9	0	0	0.9
0	0	0	0.973	0	0.973
0	0	0	0.917	0	0.918
0	0	0	0.91	0	0.91
0	0	0	0.909	0	0.909
0	0.915	0.9	0.952	0	0.999
0	0.868	0.9	0.917	0	0.998
0	0	0.9	0.974	0	0.997
0	0.807	0.9	0.518	0	0.989
0	0	0.9	0.838	0	0.983
0	0	0	0.953	0	0.954
0	0	0.9	0.501	0	0.948
0	0	0	0.921	0	0.922
0	0	0	0.903	0	0.903
0	0.963	0	0.896	0	0.996
0	0.591	0.9	0.323	0	0.969
0	0.576	0.9	0.307	0	0.968
0	0.074	0.9	0.942	0	0.938
0	0.194	0.9	0.453	0	0.929
0	0.088	0.9	0.262	0	0.926
0	0.576	0	0.808	0	0.915
0	0	0.9	0.661	0	0.964
0	0	0.9	0.56	0	0.954
0	0	0.9	0.526	0	0.95
0	0.917	0	0.716	0	0.933
0	0	0.9	0.338	0	0.931
0	0	0.9	0.208	0	0.917
0	0	0.9	0.203	0	0.916
0	0	0.9	0.196	0	0.916
0	0	0.9	0.202	0	0.916
0	0	0.9	0.504	0	0.916
0	0	0.9	0.104	0	0.906
0	0	0.9	0.087	0	0.904
0	0	0.9	0.074	0	0.903
0	0	0.9	0.064	0	0.902
0	0	0.9	0.053	0	0.901
0	0	0.9	0.044	0	0.9
0	0	0.9	0	0	0.9
0	0	0.9	0	0	0.9
0	0.915	0.9	0.909	0	0.999
0	0	0.9	0.754	0	0.974
0	0.566	0.9	0.204	0	0.962
0	0	0.9	0.539	0	0.951
0	0	0.9	0.503	0	0.948
0	0	0.9	0.509	0	0.948
0	0	0.9	0.189	0	0.915
0	0	0	0.924	0	0.924
0	0	0.9	0.222	0	0.907
0	0.566	0.9	0.51	0	0.962
0	0	0.9	0.407	0	0.912
0	0	0.9	0.265	0	0.905
0	0	0	0.984	0	0.984
0	0	0	0.975	0	0.975
0.247	0	0	0.96	0	0.968

0	0	0	0.95	0	0.95
0	0	0	0.934	0	0.934
0	0	0	0.932	0	0.932
0	0	0	0.911	0	0.912
0.974	0.985	0.9	0.996	0	0.999
0	0.912	0.9	0.917	0	0.999
0	0.988	0.8	0.508	0	0.998
0	0.98	0	0.877	0	0.997
0	0	0.9	0.942	0	0.993
0	0	0.9	0.913	0	0.99
0	0	0.9	0.912	0	0.99
0	0	0	0.988	0	0.988
0	0	0	0.979	0	0.979
0	0.576	0	0.95	0	0.978
0	0.812	0	0.856	0	0.971
0	0	0	0.964	0	0.965
0	0	0.9	0.586	0	0.956
0	0.914	0	0.454	0	0.951
0	0	0	0.948	0	0.948
0	0	0	0.942	0	0.942
0	0.564	0	0.835	0	0.925
0	0	0	0.899	0	0.9
0	0.576	0	0.868	0	0.942
0	0	0.9	0.065	0	0.902
0	0.491	0.9	0.503	0	0.972
0	0	0.9	0.81	0	0.928
0	0	0.9	0.729	0	0.926
0	0	0.9	0.659	0	0.919
0	0	0.9	0.47	0	0.915
0	0	0.9	0.535	0	0.914
0	0	0.9	0.459	0	0.913
0	0	0.9	0.439	0	0.912
0	0	0.9	0.393	0	0.91
